# Supplementary material for: Epidemiology of hypertension in Northern Tanzania: a community-based mixed-methods study
Source: BMJ Open. 2017 Nov 9;7(11):e018829. doi: 10.1136/bmjopen-2017-018829 (PMC5695455; doi:10.1136/bmjopen-2017-018829)
Supplement: Author's manuscript [file bmjopen-2017-018829.draft_revisions.pdf]

BMJ Open is committed to open peer review. As part of this commitment we make the peer review history of every article we publish publicly available.

When an article is published we post the peer reviewers' comments and the authors' responses online. We also post the versions of the paper that were used during peer review. These are the versions that the peer review comments apply to.

The versions of the paper that follow are the versions that were submitted during the peer review process. They are not the versions of record or the final published versions. They should not be cited or distributed as the published version of this manuscript.

BMJ Open is an open access journal and the full, final, typeset and author-corrected version of record of the manuscript is available on our site with no access controls, subscription charges or pay-per-view fees (http://bmjopen.bmj.com).

If you have any questions on BMJ Open's open peer review process please email editorial.bmjopen@bmj.com

EPIDEMIOLOGY OF HYPERTENSION IN NORTHERN TANZANIA: A COMMUNITY-BASED MIXED-METHODS STUDY

Journal:	*BMJ Open*
Manuscript ID	bmjopen-2017-018829
Article Type:	Research
Date Submitted by the Author:	25-Jul-2017
Complete List of Authors:	Galson, Sophie; Duke University, Division of Emergency Medicine, Department of Surgery; Duke Global Health Institute<br>Staton, Catherine; Duke University, Division of Emergency Medicine, Department of Surgery; Duke Global Health Institute<br>Karia, Frank; Kilimanjaro Christian Medical University College<br>Kilonzo, Kajiru; Kilimanjaro Christian Medical University College, Medicine<br>Lunyera, Joseph; Duke University, Division of General Internal Medicine, Department of Medicine<br>Patel, Uptal; Duke University,<br>Hertz, Julian; Duke University, Division of Emergency Medicine, Department of Surgery; Duke Global Health Institute<br>Stanifer, John; Duke University, Division of Nephrology, Department of Medicine; Duke Global Health Institute
**Primary Subject Heading**:	Global health
Secondary Subject Heading:	Cardiovascular medicine, Qualitative research, Public health, Epidemiology
Keywords:	Hypertension < CARDIOLOGY, QUALITATIVE RESEARCH, health disparities, non-communicable diseases

SCHOLARONE™ Manuscripts

EPIDEMIOLOGY OF HYPERTENSION IN NORTHERN TANZANIA: A COMMUNITY-BASED MIXED-METHODS STUDY

CORRESPONDING AUTHOR:
Sophie W Galson, MD
310 Trent Drive, Duke Box 90518
Durham, NC 27708
Sophie.Galson@duke.edu
(301)-502-7718

AUTHORS:
Sophie W Galson, MD
Division of Emergency Medicine
Department of Surgery, Duke University; Durham, NC United States
Duke Global Health Institute

Catherine A Staton, MD MSc
Division of Emergency Medicine
Department of Surgery, Duke University; Durham, NC United States
Division of Global Neurosurgery and Neuroscience, Department of Neurosurgery
Duke Global Health Institute

Francis Karia, MBA, MSc-PH
Kilimanjaro Christian Medical College; Moshi, Tanzania

Kajiru Kilonzo, MD, MMed
Department of Medicine, Kilimanjaro Christian Medical Center; Moshi, Tanzania

Joseph Lunyera, MMBS
Division of General Internal Medicine
Department of Medicine, Duke University; Durham, NC United States

Uptal Patel, MD
Duke Clinical Research Institute
Department of Medicine, Duke University; Durham, NC United States

Julian T Hertz, MD
Division of Emergency Medicine
Department of Surgery, Duke University; Durham, NC United States
Duke Global Health Institute

John W Stanifer, MD, MSc
Division of Nephrology
Department of Medicine, Duke University; Durham, NC United States
Duke Global Health Institute

WORD COUNT: 3,582

For peer review only

ABSTRACT

Introduction: Sub-Saharan Africa (SSA) is particularly vulnerable to the growing global burden of hypertension but epidemiological studies are limited and barriers to optimal management are poorly understood. Therefore, we undertook a community-based mixed-methods study in Tanzania to investigate the epidemiology of hypertension and barriers to care.

Methods: In northern Tanzania, between January 2014 and June 2015, we conducted a mixed-method study, including a cross-sectional household epidemiological survey and qualitative sessions of focus groups and in-depth interviews. For the survey, we assessed for hypertension, defined as a single blood pressure ≥ 160/100 mmHg or a two-time average of ≥ 140/90 mmHg or current use of anti-hypertensive medications. To investigate relationships with potential risk factors, we used adjusted generalized linear models. Uncontrolled hypertension was defined as a two-time average measurement of ≥ 160/100 mmHg irrespective of treatment status. Hypertension awareness was defined a self-reported disease history in a patient with confirmed hypertension. To explore barriers to care, we identified emerging themes using an inductive approach within the Framework method.

Results: We enrolled 481 adults (median age 45) from 346 households, including 123 men (25.6%) and 358 women (74.4%). Overall, the prevalence of hypertension was 28.0% (95% CI 19.4-38.7), which was independently associated with age >60 years (RR 4.68; 95% CI 2.25-9.74) and alcohol use (RR 1.76; 95% CI 1.20-2.59). Nearly half (48.3%) of the participants were aware of their disease, but almost all (95.3%) had uncontrolled hypertension. In the qualitative sessions, we identified barriers to optimal care, including poor point-of-care communication; poor understanding of hypertension; and structural barriers such as long wait-times and under-trained providers.

Conclusions: In northern Tanzania, the burden of hypertensive disease is substantial and optimal hypertension control is rare. Trans-disciplinary strategies sensitive to local practices should be explored to facilitate early diagnosis and sustained care delivery.

Keywords: Hypertension, Qualitative research, Health Disparities, Non-communicable diseases

For peer review only

STRENGTHS AND LIMITATIONS OF THIS STUDY

- Rigorous study design based on random, community-based sampling.
- The mixed-methods approach allowed for triangulation from multiple data sources with reproducible methods.
- Barriers to optimal hypertension care were explored using qualitative studies with key informants from both biomedicine and traditional medicine practices.
- As this was a cross-sectional study, causal inferences cannot be drawn.
- Our role as biomedical practitioners may have limited our ability to interpret results (researcher bias) about differences in disease understanding, and our inferences are derived from a biomedical perspective.

INTRODUCTION

Non-communicable diseases (NCDs), including hypertension, are a global epidemic disproportionately affecting health outcomes in low-and middle-income countries (LMICs) [1-3]. In sub-Saharan Africa (SSA) alone, more than 125 million people are expected to have hypertension by the year 2025 [4, 5]. Hypertensive-related complications are currently one of the leading causes of morbidity in SSA, and by 2030 hypertension and other NCDs are projected to surpass communicable diseases as the top cause of mortality [2, 3, 6]. Despite the overwhelming burden, SSA is mostly unprepared to address this impending public health crisis [7, 8].

Previously-identified barriers to addressing hypertension in SSA include under-recognition, under-treatment, and a limited understanding of its epidemiology [9, 10]. In particular, the lack of reliable health statistics and a paucity of community-based epidemiological data limit the ability for detection, surveillance, and creation of public health strategies for prevention and treatment [8, 11]. In northern Tanzania, for example, only 10-20% of patients with previously detected hypertension are receiving treatment, and only 16% of those on treatment were adequately controlled [12, 13].

Differences in hypertension care are related to several factors beyond healthcare access alone, including limited health literacy, cultural and social barriers, and heuristically different health belief models [14]. As such, understanding the epidemiology of hypertension as well as the social and community barriers to optimal care is critical for developing prevention and treatment strategies; however, few such studies have been conducted. Therefore, as part of the Comprehensive Kidney Disease Assessment for Risk Factors, Epidemiology, Knowledge, and Attitudes (CKD-AFRiKA) study, we conducted a mixed-methods study in order to characterize the community-based epidemiology of hypertension and barriers to optimal care through exploration of patient- and community-centered perspectives [15, 16].

METHODS

Ethics Statement

The study protocol was approved by Duke University Institutional Review Board (#Pro00040784), the Kilimanjaro Christian Medical College (KCMC) Ethics Committee (EC#502), and the National Institute for Medical Research

(NIMR) in Tanzania. Written informed consent (by signature or thumbprint) was obtained from all participants, and all participants with abnormal findings received counseling, educational pamphlets, and reimbursement with referral for follow-up.

Study Setting

We conducted a mixed methods study between December 2013 and June 2015 in the Kilimanjaro Region of Tanzania. The adult regional population is greater than 900,000 people, and it has a female majority (58%)[17]. Almost 35% of the adult population lives in an urban setting, which is comparable to national estimates, and the HIV prevalence is 3-5% [17, 18]. The unemployment rate is 19%, and most people have only a primary education (77%) [19]. The median age, average household size, and occupation distribution are similar to national estimates [17-19]. The largest ethnic group is the Chagga tribe, and Swahili is the major language [17]. The region comprises seven districts; our study was conducted in the Moshi Urban and Moshi Rural districts [17].

Quantitative Sampling and Data Collection

Detailed sampling methods for the CKD-AFRiKA Study have been previously reported [15, 16, 20]. In brief, a three-stage cluster probability sampling method, stratified by urban and rural setting, was used to randomly select neighborhoods based on probability proportional to size. Within each selected neighborhood, a cluster site was determined using geographic points randomly generated by Arc Global Information Systems (ArcGIS), v10.2.2 (Environmental Systems Research Institute, Redlands, CA). From the cluster site, households were then randomly chosen based on both a coin-flip and die-rolling technique according to our established protocol [15].

All non-pregnant, community-dwelling adults (age ≥ 18 years old) from the selected households were recruited into the study. The sample size was designed to estimate the prevalence of chronic kidney disease with a precision of 5% when accounting for the cluster-design effect. To reduce non-response rates, a minimum of two additional visits were attempted during off-hours (evenings and weekends) as well as multiple phone calls using mobile phone numbers.

All data were collected using trained, local surveyors. Each participant completed a demographic and medical history survey, which included self-reported history of diabetes, hypertension, HIV, kidney disease, and heart disease (coronary, structural, or heart failure). If participants were receiving biomedical treatment in the form of medical therapy, specific drug information was collected. Women additionally gave a self-reported history for pregnancy or menstruation. Awareness was defined as giving a self-reported history of hypertension and subsequently testing positive for hypertension in our screening process.

Anthropometric data (including height, weight, and body mass index) were recorded for each participant. Normal weight was defined as a BMI of 20 to 24.9 kg/m^2. Overweight was defined as a BMI ≥25 kg/m^2 and obesity was defined as a BMI ≥30 kg/m^2. We measured blood pressure using the automated Omron HEM-712 sphygmomanometer (Omron Healthcare, Inc.; Bannockburn, IL) that has an adjustable cuff size. The machine was calibrated monthly during data collection. All participants were seated in an erect position with feet flat on the floor for a minimum of five minutes before measurements. Hypertension was defined as a single blood pressure measurement of greater than 160/100 mmHg, a two-time average measurement of greater than 140/90 mmHg, or current self-reported use of anti-hypertensive medications. Uncontrolled hypertension was defined as a two-time average measurement of greater than 160/100 mmHg irrespective of treatment status.

For peer review only - http://bmjopen.bmj.com/site/about/guidelines.xhtml

Qualitative Data Collection

To explore patient- and community-centered perspectives related to barriers in optimal hypertension care, we conducted focus group discussions (FGDs) and in-depth interviews in a central, easily accessible location. These sessions have been described previously [21]. In brief, we conducted FGDs and in-depth interviews with key informants from the community including well-adults from the general population, chronically-ill adults receiving care at the hospital medicine clinics, adults receiving care from traditional healers, adults purchasing traditional medicines from herbal vendors, traditional healers, herbal vendors, and medical doctors. All sessions were semi-structured, open-ended, and probing. The discussion guide was initially written in English and then translated to Swahili by an independent team. All sessions were moderated by a native, local member of our team (FK). All sessions were audio-recorded, and two note-takers transcribed and independently translated each session. A moderator then reviewed the transcripts to ensure accuracy. Debriefings were held after each session, and team meetings were again held following translation.

Quantitative Data Analysis

The mean and standard deviation (SD) or median and inter-quartile ranges (IQR) were reported for continuous variables. Prevalence estimates were sample-balanced using age- and gender-weights based on the 2012 urban and rural district-level census data. We used a Chi-squared test or Fisher's exact test to compare differences between groups. All p-values are two-sided at a 0.05 significance level. Quantitative data were analyzed using STATAv.14 (STATA Corp., College Station, TX).

A secondary aim of the analysis was to explore associations between hypertension and potential risk factors related to lifestyle. Crude and adjusted prevalence risk ratios (PRR) were estimated using generalized linear models with a log link, and we used Taylor Series linearization to account for the design effect on variance due to cluster sampling. Separate uni-variable and multi-variable models were fitted to hypertension status for each lifestyle-related variable including alcohol use, tobacco use, traditional medicine use, living in an urban environment, and overweight/obesity status. Models were adjusted for confounding factors potentially associated with hypertension and each potential risk factor, including age, gender, and ethnicity. We did not include education or occupation in our models due to *a priori* assumptions about their potential upstream causal association with lifestyle-related risk factors.

All quantitative data were collected on paper and then electronically entered into and managed using REDCap electronic data capture tools hosted at Duke University [22]. All data were verified after electronic data entry by an independent reviewer to ensure accuracy.

Qualitative Data Analysis

We conducted a thematic analysis of the qualitative data by applying an inductive approach to the framework method [23]. The approach was based, in part, on our previously-developed model which explored determinants of traditional medicine use and biomedical healthcare utilization among individuals in Kilimanjaro with NCDs, including hypertension [21]. After data reduction, we performed open-coding of all transcripts. We used a 'cultural insider' (emic) and a 'cultural outsider' (etic) to independently code the data. The cultural insider was a native researcher living in the region (FK) and the cultural outsider was a researcher foreign to the region (JWS). Comparisons were made between each code set and areas of disagreement were discussed and resolved by revisiting the data. This approach allowed us to explore concepts that otherwise may have been overlooked or misinterpreted by either researcher individually. The qualitative coding, analytic memos, and corresponding

For peer review only

matrices were stored and analyzed using NViVOv.10.0 (QRS International Pty Ltd, Melbourne, Australia). The codes were grouped together into categories, and we used a coding index to formulate connections and explore relationships.

RESULTS

Table 1. Baseline Characteristics for the Quantitative Study

Variable (n, %)	Total (n=481)	Normotensive (n=332)	Hypertensive (n=149)	p-value
**Gender**				0.12
Male	123 (25.6%)	78 (23.5%)	45 (30.2%)	
Female	358 (74.4%)	254 (76.5%)	104 (69.8%)	
**Age**				<0.01
18-39 years old	172 (35.8%)	152 (45.8%)	20 (13.4%)	
40-59 years old	191 (39.7%)	132 (39.8%)	59 (39.6%)	
60+ years old	118 (24.5%)	48 (14.5%)	70 (47.0%)	
**Ethnicity**				0.40
Chagga	288 (59.9%)	193 (58.1%)	95 (63.7%)	
Pare	66 (13.7%)	51 (15.4%)	15 (10.1%)	
Sambaa	27 (5.6%)	20 (6.0%)	7 (4.7%)	
Other[§]	100 (20.8%)	68 (20.5%)	32 (21.5%)	
**Education**				<0.01
None	31 (6.4%)	11 (3.31%)	20 (13.4%)	
Primary	349 (72.6%)	246 (74.1%)	103 (69.1%)	
Secondary	74 (15.4%)	54 (16.3%)	20 (13.4%)	
Post-Secondary	27 (5.6%)	21 (6.3%)	6 (4.03%)	
**Occupation**				<0.01
Unemployed[#]	74 (15.4%)	55 (16.6%)	19 (12.8%)	
Farmer/Wage Earner	199 (41.4%)	135 (40.7%)	64 (43.0%)	
Small Business/Vendors	158 (32.8%)	121 (36.5%)	37 (24.8%)	
Professional[†]	50 (10.4%)	21 (6.3%)	29 (19.5%)	
**Lifestyle Practices**				
Ongoing tobacco use	50 (10.4%)	34 (10.2%)	16 (10.7%)	0.87
Ongoing alcohol use	198 (41.2%)	121 (36.4%)	77 (51.7%)	0.02
Traditional medicine use	272 (56.6%)	196 (59.0%)	76 (51.0%)	0.10
**Self-Reported Medical History**				
Diabetes	61 (12.7%)	29 (8.7%)	32 (21.5%)	<0.01
Hypertension	134 (28.0%)	62 (18.8%)	72 (48.3%)	<0.01
Stroke	8 (1.7%)	2 (0.6%)	6 (4.0%)	0.01
Heart Disease[*]	18 (3.7%)	7 (2.1%)	7 (4.7%)	0.08
Kidney Disease	14 (2.9%)	10 (3.0%)	4 (2.7%)	0.84

Table 2. Baseline Characteristics for the Qualitative Study

	FGD1	FGD2	FGD3	FGD4	FGD5	In-Depth Interviews
Study Population	Clinic Patients	General Population	Clinic Patients	General Population	Medical Doctors	Patients from Healers and Vendors
**Participants (N)**	15	12	16	12	4	11
**Gender**						
Male	0 (0%)	0(0%)	16(100%)	12(100%)	2(50%)	5(45%)
Female	15(100%)	12(100%)	0(0%)	0(0%)	2(50%)	6(55%)
Age range (years)	25-61	26-65	18-70	18-74	30-36	19-60
**Ethnicity**						
Chagga	11 (73%)	9 (75%)	11 (69%)	4 (33%)	2 (50%)	2 (18%)
Pare	2 (13%)	2 (17%)	2 (13%)	5 (42%)	0	0
Maasai	0	0	0	0	0	4 (36%)
Sambaa	1 (7%)	1 (8%)	1 (6%)	0	0	3 (27%)
Other*	1 (7%)	0	2 (13%)	3 (25%)	2 (50%)	2 (18%)
**Education**						
None	0	0	0	0	0	2 (18%)
Primary	11 (73%)	10 (83%)	10 (63%)	3 (25%)	0	4 (36%)
Secondary	3 (20%)	2 (17%)	5 (31%)	6 (50%)	0	1 (9%)
University	1 (7%)	0	1 (6%)	3 (25%)	4 (100%)	4 (36%)
**Occupation**						
Unemployed[#]	2 (13%)	4 (33%)	0	1 (8%)	0	3 (27%)
Student	0	0	4 (25%)	5 (42%)	0	0
Farmer/Wage Earner	4 (27%)	3 (25%)	8 (50%)	3(25%)	0	5 (45%)
Small Business	3 (20%)	2 (17%)	3 (19%)	2 (17%)	0	1 (9%)
Professional[†]	4 (27%)	3 (25%)	1 (6%)	1 (8%)	4(100%)	2 (18%)
**Religion**						
Roman Catholic	5 (33%)	5 (42%)	8 (50%)	1 (8%)	3 (75%)	7 (64%)
Lutheran	6 (40%)	4 (33%)	4 (25%)	2 (17%)	0	1 (9%)
Christian Evangelical	1 (7%)	1 (8%)	2 (13%)	5 (42%)	1 (25%)	1 (9%)
Christian (Other)	2 (13%)	0	0	0	0	0
Islam	1 (7%)	2 (17%)	2 (13%)	4 (33%)	0	2 (18%)

Study Populations

We enrolled 481 adults into the quantitative study (**Table 1**). The median age was 45.0 years (IQR 35–59). The majority of participants were women (n = 358; 74%), lived in an urban location (n = 370; 77%), ethnically Chagga (n = 288; 60%), and only had a primary school education (n = 349; 73%). The most common occupation among participants was farming or daily wage work (n =199; 41%). Many participants reported ongoing use of alcohol (n=198; 41%) and traditional medicine use over the previous year (n=272; 57%).

The household non-response rate was 15.0% and the individual non-response rate was 20.6%. Compared with the regional population [17], men ($p<0.001$) and young adults 18–39 years old ($p = 0.001$) were more likely to be non-responders in our study, and the proportion of participants with a secondary or post-secondary education (22%) was higher than the regional average (15%) ($p = 0.02$). We observed no significant differences in occupation between the responders and non-responders ($p = 0.64$).

In the qualitative study, we conducted five FGDs and 11 in-depth interviews (**Table 2**). FGDs and in-depth interviews included even numbers of men (n= 35; 50%), and women (n=35; 50%) and had an age range of 18 to 74 years. Most participants were of the Chagga ethnic group (n=37; 53%), and were Roman Catholic (n=29; 41%), but Islamic (n=11; 16%), Lutheran (n=17; 24%), and Christian evangelical (n=11; 16%) were also represented as well as thirteen different tribal ethnicities. Education levels varied from none (n=2; 3%) to university level (n=13; 19%), but the majority had only completed a primary education (n=38; 54%).

Burden of Hypertension

The prevalence of hypertension was 28% (95% CI 19.4-38.7). The design effect of the cluster sampling was 2.34, with a neighborhood-level ICC coefficient of 0.075. Mean systolic blood pressure was 129.5 mmHg (SD 24.3), mean diastolic blood pressure was 77.6 mmHg (SD 12.2), and the median age was 58 years (IQR 45-65). The median BMI of individuals with hypertension was 27.4 kg/m^2 (IQR 24-30). Participants with hypertension were more likely to be men, older, report ongoing alcohol use, live in an urban environment, have less education, and be employed as professionals (p<0.05 for all) (**Table 1**).

Crude and adjusted prevalence risk ratios for the relation between lifestyle-related factors and hypertension are reported in **Table 3**. In crude models, traditional medicine use was inversely associated with hypertension prevalence (PRR 0.60; 95% CI 0.41-0.87), and alcohol use was significantly associated with higher prevalence of hypertension (PRR 2.29; 95% 1.26-4.15). These associations remained significant even after adjustment for age, gender, and ethnicity, with a PRR 0.37 (95% CI 0.26-0.54) and PRR 1.72 (95% CI 1.15-2.58) for traditional medicine use and alcohol use, respectively. We did not observe an association between hypertension prevalence and obesity, urban residence, or tobacco use (p>0.05 for all).

Table 3. Associations between lifestyle factors and hypertension; CKD-AFRIKA, 2015.

Variables	Prevalence Risk Ratios (95% CI)	
	Unadjusted	Adjusted*
Ongoing Tobacco Use	1.25 (0.75, 2.10)	0.68 (0.41, 1.14)
Traditional Medicine Use	**0.60 (0.41, 0.87)**	**0.37 (0.26, 0.54)**
Ongoing Alcohol Use	**2.29 (1.26, 4.15)**	**1.72 (1.15, 2.58)**
Overweight/Obese	1.00 (0.57, 1.74)	1.28 (0.84, 1.97)
Urban Residence	0.58 (0.34, 1.00)	0.85 (0.59, 1.22)

*Adjusted for age, gender, and ethnicity
BOLD: Significant at the 5% level

Barriers to Optimal Care

Despite the high disease burden, only half of participants with elevated blood pressure (48%) were aware of having hypertension. Few (23%) reported taking biomedicines for hypertension, and 12% reported taking both biomedicines and traditional medicines. Almost all (95%) had uncontrolled hypertension. A major theme that emerged as an important barrier for awareness and disease self-management was a difference in individual chronic disease understanding. Most notably, we identified quality or perceived quality of the biomedical healthcare delivery, disease expression, chronicity of disease, and traditional health belief models as important

For peer review only

contributors to the observed differences in chronic disease understanding, which itself contributed to unrealistic expectations of cure, perceived treatment failures, and medical non-compliance (**Figure 1**).

Participants related structural issues and point-of-care communication issues as key barriers to optimal biomedical healthcare delivery. Structural issues in the delivery of biomedical healthcare, including long wait times, under-staffing, lack of experience by healthcare providers, and medication costs, were particularly concerning, and together with poor point-of-care communication between patient and provider, appeared to substantially contribute to differences in chronic disease understanding through unrealistic expectations of cure expressed as concern over ineffective or inappropriate therapies:

> *MDs have a lot of patients to take care of so they lack enough time to give explanations. They hurry so that they can save as many patients as possible.*

> *I attended the hospital and treatment was begun but with no success for a long time; instead other parts in my body began to swell. At that time I decided to discharge myself from the hospital, and my grandmother gave me local herbals which cured me.*

For many participants, differences in disease understanding were also closely related to the disease expression or the symptom complex of disease. For chronic, generally asymptomatic diseases such as hypertension, this led to unrealistic expectations of cure, greater perceptions of treatment failure by biomedicines, and increased medical non-compliance:

> *You know you have a disease because the body always has symptoms.*

> *You know you are healed as you do not have to attend the hospital anymore because your symptoms have disappeared.*

For chronic diseases such as hypertension, chronicity or duration of disease was an especially salient topic closely related to differences in disease understanding. Chronic diseases were understood to be diseases that have either been untreated or undertreated, and even infectious diseases were viewed as chronic when left untreated:

> *Anything that stays in the human body for a long time without being cured, like amoebae and bilharzias* [schistosomiasis] *is a chronic disease.*

From the biomedical perspective, this different understanding of chronicity led to many challenges in achieving optimal care for patients with hypertension, particularly with respect to medication compliance, expectations of cure, and perceived treatment failure. As biomedical doctors explained:

> *Some patients with hypertension, after their blood pressure is controlled, they then believe they are cured after 2 or 3 months. They go to follow-up and see that their blood pressure normal; therefore, they assume they are cured and stop their medications. The chronicity of the disease they do not understand well.*

For peer review only

…the people keep on seeking a 'cure' for something that is a chronic disease.

Finally, traditional health belief models were also closely associated with differences in disease understanding. Even for chronic diseases such as hypertension, participants expressed the importance of traditional medicines, and community elders and family members were considered important sources of healthcare knowledge.

My family and I prefer not to go to hospitals. My grandparents taught us a lot (especially about plant roots) about healing and curing… my father still will not use any hospital medicines.

…most of the chronic diseases are cured by traditional medicines.

Discussion

In a community-based setting in northern Tanzania, we found an alarmingly high burden of hypertension. The prevalence observed was 10-20% higher than similar regions in SSA and comparable with several upper and middle-income countries, including South Africa, Brazil, and China [2, 4, 5, 8, 10, 13, 24]. Despite the high burden of hypertension, awareness was low and few had achieved optimal blood pressure control, which may be explained by observed gaps in communication, quality of healthcare delivery, and traditional medicine health belief models. In particular, we identified differences in disease understanding as they relate to a disease expression through symptom complexes, disease chronicity, and traditional health beliefs as potentially important barriers for achieving optimal hypertension care.

As epidemiological transitions reshape the region, Tanzania is at great risk for an explosive growth in the burden of NCDs, including hypertension [8, 25, 26]. The rapid pace of urbanization and economic growth are accelerating the rate of this transition; thus, as evidenced by the high prevalence we are already observing, there is an urgent need for action [5]. Aggressive efforts should be made to diagnose and capture hypertensive patients at every single interaction within health systems. Considering the low rates of awareness in our study, all settings for diagnosis and delivery of healthcare should be explored including community centers, traditional medicine providers and emergency departments (EDs). Community-centered care models may be beneficial to reduce risk factors, improve treatment adherence, and have been successful previously in SSA [7, 27]. Given the important role of traditional medicine practices, partnership with traditional medicine providers should be considered to assist with risk factor reduction and care coordination, including early referral [28]. Additionally, ED-based screening has also been successful at capturing undiagnosed/uncontrolled hypertension cases and linking patients to care in high-income countries; yet ED-based care models for hypertension have not been widely explored in SSA [29-31]. For example, ED-based interventions to screen for hypertension and modifiable risk factors as the first step of a care pathway may prove to be highly effective in hypertension care. Beyond diagnosis, careful consideration of the local environment and barriers to care will be necessary to create successful educational programs and sustain hypertension control. Educational interventions should focus on the concepts of chronicity and disease expression, and incorporate traditional health beliefs. Targeted and culturally-tailored engagement with patients may prevent poor hypertension outcomes through improved self-management, particularly with medication compliance, expectations of cure, and perceived treatment success [32].

We found traditional medicines use was associated with lower prevalence of hypertension. This may indicate a protective effect or alternatively, a form of selection bias in the sampling process but stresses the importance of

For peer review only

culturally competent interventions when addressing hypertension. In contrast, we found alcohol to be positively associated with hypertension in this population, consistent with trends throughout SSA [33-35]. This region of Tanzania has a particularly high prevalence of alcohol use due to cultural acceptability and home-brewing culture [36]. Given the high burden of alcohol, practitioners in all locations should be cognizant of this risk factor during both hypertension screening and treatment.

Our study was unique in exploring hypertension in the Kilimanjaro area by utilizing a rigorous randomly-sampled, household-level survey as part of a mixed-method design that also included qualitative sessions with key informants. We explored latent themes and social/community context for treatment failure, and by leveraging this thematic analysis we were able to identify targetable barriers to optimal hypertension diagnosis, treatment, and control. Nonetheless, we noted potential limitations to our study. First, selection bias from non-response may be present. To address any non-response bias that may have arisen from differences between the respondents and non-respondents, we used sample-balanced weights for age and gender and explored differences in occupation and education level between the two groups. In regards to internal validity, we only measured blood pressure at one setting; however, two measurements separated by > 5 minutes were performed and previous studies have shown that sustained elevated blood pressure in one setting may be sensitive to establish a diagnosis of hypertension [30]. Misclassification of disease around the cutoff points for hypertension may also be present although we expect this misclassification to be non-differential. Additionally, as this was a cross-sectional study, causal inferences cannot be drawn and associations may be influenced by confounding from unmeasured variables. Our role as biomedical practitioners may have limited our ability to interpret results (researcher bias) about differences in disease understanding, and our inferences are derived from a biomedical perspective. Additionally, although we used insider-outsider coding, local non-medical surveyors, and local moderators for qualitative data collection, reporting bias may still be present.

In a community-based sample of adults from the Kilimanjaro region of northern Tanzania we observed a high prevalence of hypertension, most of which was uncontrolled. Alcohol use may be an important risk factor for hypertension, and we identified several emerging cultural and social themes as barriers to optimal hypertension care, including most notably difference in disease understanding related to quality of healthcare delivery, chronicity of disease, disease expression, and traditional belief models. Hypertension care-models will need to leverage all existing resources from the ED, to community centers, to traditional healers in order to address the growing burden of hypertension in the region, and future studies are needed to develop targeted, culturally-tailored interventions designed to improve hypertension disease understanding.

ACKNOWLEDGMENTS

We would like to thank Professors G. Ralph Corey and John Bartlett, and all the staff of the KCMC-Duke Collaboration in Moshi, Tanzania for all of their efforts on the Comprehensive Kidney Disease Assessment For Risk factors, epidemiology, Knowledge, and Attitudes (CKD AFRiKA) Study. We give a special thanks to Carol Sangawe, Cynthia Asiyo, Nicola West, and Edith Aloyce Macha for their integral role implementing the study and Jeffrey Hawley and Audrey Brown at the Duke Office of Clinical Research for their help in data management. We are also grateful to Estomih Mduma and his team for their help with Swahili translation needs.

CONTRIBUTION

JS and UP developed the concept of the project. JS and FK conducted data collection. SG and JS contributed to writing the manuscript. JS and JL were responsible for the analysis plan and data analysis. CS, JS, SG, JL, KK, JH, UP, and FK were responsible for the final editing and all authors approved the final manuscript.

For peer review only

COMPETING INTERESTS

All authors have completed the ICMJE uniform disclosure form at www.icmje.org/coi_disclosure.pdf and declare: no support from any organization for the submitted work; no financial relationships with any organizations that might have an interest in the submitted work in the previous three years; no other relationships or activities that could appear to have influenced the submitted work.

FUNDING

This study was supported by an NIH Research Training Grant (#R25 TW009337) funded by the Fogarty International Center and the National Institute of Mental Health; and a Research and Prevention Grant funded by the International Society of Nephrology Global Outreach Committee. Dr. Staton would like to acknowledge salary support funding from the Fogarty International Center (Staton, K01 TW010000-01A1).

DATA SHARING

We have concerns about the ethics of openly releasing the entire dataset to the public as the structure of the dataset would result in loss of participant anonymity. However, we will ensure that the dataset is openly available to researchers who contact us and meet confidentiality requirements (documentation of ethics training in conduct of human-subject research). They may contact Dr. John W Stanifer, DCRI 2400 Pratt St, Durham NC 27710 or john.stanifer@duke.edu

FIGURE LEGEND

Fig. 1 Conceptual model describing the hypothesized relationship between disease understanding and hypertension outcomes.

For peer review only

REFERENCES

1. Abegunde DO, Mathers CD, Adam T, et al. The burden and costs of chronic diseases in low-income and middle-income countries. *Lancet (London, England)*. 2007;370(9603):1929-38.
2. WHO | A global brief on hypertension. *WHO*. 2013.
3. Institute for Health Metrics and Evaluation (IHME) 2015 [Available from: http://vizhub healthdata org/gbd-compare.
4. Pastakia SD, Ali SM, Kamano JH, et al. Screening for diabetes and hypertension in a rural low income setting in western Kenya utilizing home-based and community-based strategies. Globalization and health. 92013. p. 21.
5. Twagirumukiza M, De Bacquer D, Kips JG, et al. Current and projected prevalence of arterial hypertension in sub-Saharan Africa by sex, age and habitat: an estimate from population studies. *Journal of hypertension*. 2011;29(7):1243-52.
6. Whitworth JA. 2003 World Health Organization (WHO)/International Society of Hypertension (ISH) statement on management of hypertension. *Journal of hypertension*. 2003;21(11):1983-92.
7. Twagirumukiza M, Van Bortel LM. Management of hypertension at the community level in sub-Saharan Africa (SSA): towards a rational use of available resources. *Journal of human hypertension*. 2011;25(1):47-56.

8. Peck R, Mghamba J, Vanobberghen F, et al. Preparedness of Tanzanian health facilities for outpatient primary care of hypertension and diabetes: a cross-sectional survey. *Lancet Glob Health*. 2014;2(5):e285-92.
9. Naanyu V, Vedanthan R, Kamano JH, et al. Barriers Influencing Linkage to Hypertension Care in Kenya: Qualitative Analysis from the LARK Hypertension Study. *Journal of general internal medicine*. 2016;31(3):304-14.
10. Chow CK, Teo KK, Rangarajan S, et al. Prevalence, awareness, treatment, and control of hypertension in rural and urban communities in high-, middle-, and low-income countries. *Jama*. 2013;310(9):959-68.
11. Churchill LO. Epidemiology of ischaemic heart disease in sub-Saharan Africa. *Cardiovasc J Afr*. 2013;24(2):34-42.
12. Dewhurst MJ, Dewhurst F, Gray WK, et al. The high prevalence of hypertension in rural-dwelling Tanzanian older adults and the disparity between detection, treatment and control: a rule of sixths? *Journal of human hypertension*. 2012;27(6):374-80.
13. Mosha NR, Mahande M, Juma A, et al. Prevalence,awareness and factors associated with hypertension in North West Tanzania. *Global health action*. 2017;10(1):1321279.
14. Nnko S, Bukenya D, Kavishe BB, et al. Chronic Diseases in North-West Tanzania and Southern Uganda. Public Perceptions of Terminologies, Aetiologies, Symptoms and Preferred Management. *PloS one*. 2015;10(11):e0142194.
15. Stanifer JW, Maro V, Egger J, et al. The epidemiology of chronic kidney disease in Northern Tanzania: a population-based survey. *PloS one*. 2015;10(4):e0124506.
16. Stanifer JW, Egger JR, Turner EL, et al. Neighborhood clustering of non-communicable diseases: results from a community-based study in Northern Tanzania. *BMC Public Health*. 2016;16:226.
17. Central Census Office and National Bureau of Statistics. United Republic of Tanzania: 2012 Population and Housing Census 2013 [Available from: http://www.nbs.go.tz. .
18. Demographic and Health Surveys. Program. United Republic of Tanzania HIV/AIDS and Malaria Indicator Survey 2011-2012. 2013 [Available from: http://dhsprogram.com/pubs/pdf/AIS11/AIS11.pdf.
19. Development Committee. United Republic of Tanzania: Education Sector Performance Report, 2010-2011 2013 [Available from: http://www.ed-dpg.or.tz.
20. Stanifer JW, Cleland CR, Makuka GJ, et al. Prevalence, Risk Factors, and Complications of Diabetes in the Kilimanjaro Region: A Population-Based Study from Tanzania. *PloS one*. 2016;11(10):e0164428.
21. Stanifer JW, Patel UD, Karia F, et al. The determinants of traditional medicine use in Northern Tanzania: a mixed-methods study. *PloS one*. 2015;10(4):e0122638.
22. Harris PA, Taylor R, Thielke R, et al. Research electronic data capture (REDCap)--a metadata-driven methodology and workflow process for providing translational research informatics support. *Journal of biomedical informatics*. 2008;42(2):377-81.
23. Gale NK, Heath G, Cameron E, et al. Using the framework method for the analysis of qualitative data in multi-disciplinary health research. *BMC medical research methodology*. 2013;13:117.
24. Kearney PM, Whelton M, Reynolds K, et al. Worldwide prevalence of hypertension: a systematic review. *Journal of hypertension*. 2004;22(1):11-9.
25. Yikona J. Non-communicable disease in sub-Saharan Africa. *Lancet (London, England)*. 2001;357(9249):74.
26. Mufunda J, Chatora R, Ndambakuwa Y, et al. Emerging non-communicable disease epidemic in Africa: preventive measures from the WHO Regional Office for Africa. *Ethnicity & disease*. 2006;16(2):521-6.
27. Jeffries D. Risk in cardiovascular disease. Having so many different guidelines about reducing risk is confusing. *BMJ (Clinical research ed)*. 2000;321(7254):175.
28. Liwa AC, Smart LR, Frumkin A, et al. Traditional Herbal Medicine Use Among Hypertensive Patients in Sub-Saharan Africa: A Systematic Review. *Curr Hypertens Rep*. 2014;16(6):437.
29. Chernow SM, Iserson KV, Criss E. Use of the emergency department for hypertension screening: a prospective study. *Annals of emergency medicine*. 1987;16(2):180-2.

For peer review only

30. Ackerson HDB, Linda D, Lynn. Reproducibility of increased blood pressure during an emergency department or urgent care visit. *Annals of emergency medicine*. 2003;41(4):507-12.
31. Tan N, Taylor DM. Feasibility and outcomes of screening for cardiovascular risk factors in the emergency department. *Emergency medicine Australasia : EMA*. 2013;25(2):175-81.
32. Warsi A WP, LaValley MP, Avorn J, Solomon DH. Self-management education programs in chronic disease: a systematic review and methodological critique of the literature. *Arch Intern Med*.9-23;164(15):1641-9.

33. Xin X, He J, Frontini MG, et al. Effects of alcohol reduction on blood pressure: a meta-analysis of randomized controlled trials. *Hypertension*. 2001;38(5):1112-7.

34. He J, Bazzano LA. Effects of lifestyle modification on treatment and prevention of hypertension. *Current opinion in nephrology and hypertension*. 2000;9(3):267-71.

35. Nahimana MR, Nyandwi A, Muhimpundu MA, et al. A population-based national estimate of the prevalence and risk factors associated with hypertension in Rwanda: implications for prevention and control. *BMC Public Health*. 2017;18(1):2.
36. Mitsunaga T, Larsen U. Prevalence of and risk factors associated with alcohol abuse in Moshi, northern Tanzania. *Journal of biosocial science*. 2008;40(3):379-99.

For peer review only

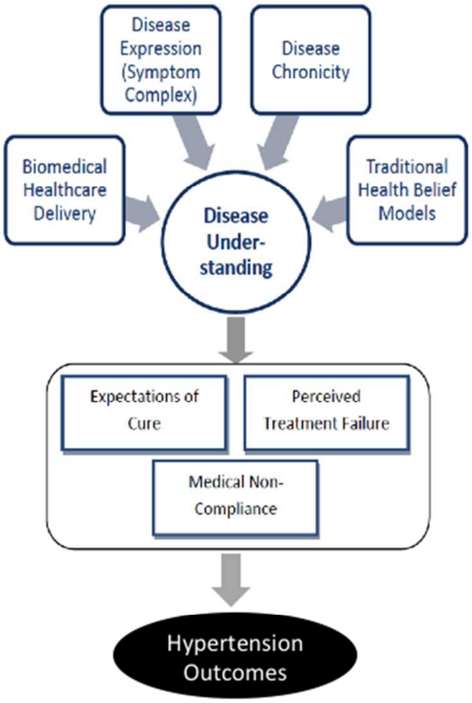

Conceptual model describing the hypothesized relationship between disease understanding and hypertension outcomes.

254x190mm (72 x 72 DPI)

STROBE 2007 (v4) Statement—Checklist of items that should be included in reports of *cross-sectional studies*

Section/Topic	Item #	Recommendation	Reported on page #
**Title and abstract**	1	(*a*) Indicate the study's design with a commonly used term in the title or the abstract	1
		(*b*) Provide in the abstract an informative and balanced summary of what was done and what was found	2
**Introduction**			
Background/rationale	2	Explain the scientific background and rationale for the investigation being reported	3
Objectives	3	State specific objectives, including any prespecified hypotheses	3
**Methods**			
Study design	4	Present key elements of study design early in the paper	4
Setting	5	Describe the setting, locations, and relevant dates, including periods of recruitment, exposure, follow-up, and data collection	4
Participants	6	(*a*) Give the eligibility criteria, and the sources and methods of selection of participants	4
Variables	7	Clearly define all outcomes, exposures, predictors, potential confounders, and effect modifiers. Give diagnostic criteria, if applicable	4
Data sources/ measurement	8*	For each variable of interest, give sources of data and details of methods of assessment (measurement). Describe comparability of assessment methods if there is more than one group	4
Bias	9	Describe any efforts to address potential sources of bias	*4*
Study size	10	Explain how the study size was arrived at	4
Quantitative variables	11	Explain how quantitative variables were handled in the analyses. If applicable, describe which groupings were chosen and why	5
Statistical methods	12	(*a*) Describe all statistical methods, including those used to control for confounding	5
		(*b*) Describe any methods used to examine subgroups and interactions	5
		(*c*) Explain how missing data were addressed	5
		(*d*) If applicable, describe analytical methods taking account of sampling strategy	5
		(*e*) Describe any sensitivity analyses	NA
**Results**			

For peer review only

Participants	13*	(a) Report numbers of individuals at each stage of study—eg numbers potentially eligible, examined for eligibility, confirmed eligible, included in the study, completing follow-up, and analysed	4
		(b) Give reasons for non-participation at each stage	4
		(c) Consider use of a flow diagram	4 (reference)
Descriptive data	14*	(a) Give characteristics of study participants (eg demographic, clinical, social) and information on exposures and potential confounders	6
		(b) Indicate number of participants with missing data for each variable of interest	4
Outcome data	15*	Report numbers of outcome events or summary measures	6,7
Main results	16	(*a*) Give unadjusted estimates and, if applicable, confounder-adjusted estimates and their precision (eg, 95% confidence interval). Make clear which confounders were adjusted for and why they were included	6,7
		(*b*) Report category boundaries when continuous variables were categorized	4
		(*c*) If relevant, consider translating estimates of relative risk into absolute risk for a meaningful time period	NA
Other analyses	17	Report other analyses done—eg analyses of subgroups and interactions, and sensitivity analyses	8
**Discussion**			
Key results	18	Summarise key results with reference to study objectives	10
Limitations	19	Discuss limitations of the study, taking into account sources of potential bias or imprecision. Discuss both direction and magnitude of any potential bias	11
Interpretation	20	Give a cautious overall interpretation of results considering objectives, limitations, multiplicity of analyses, results from similar studies, and other relevant evidence	10
Generalisability	21	Discuss the generalisability (external validity) of the study results	11
**Other information**			
Funding	22	Give the source of funding and the role of the funders for the present study and, if applicable, for the original study on which the present article is based	11

*Give information separately for cases and controls in case-control studies and, if applicable, for exposed and unexposed groups in cohort and cross-sectional studies.

Note: An Explanation and Elaboration article discusses each checklist item and gives methodological background and published examples of transparent reporting. The STROBE checklist is best used in conjunction with this article (freely available on the Web sites of PLoS Medicine at http://www.plosmedicine.org/, Annals of Internal Medicine at http://www.annals.org/, and Epidemiology at http://www.epidem.com/). Information on the STROBE Initiative is available at www.strobe-statement.org.

For peer review only

BMJ Open

EPIDEMIOLOGY OF HYPERTENSION IN NORTHERN TANZANIA: A COMMUNITY-BASED MIXED-METHODS STUDY

Journal:	*BMJ Open*
Manuscript ID	bmjopen-2017-018829.R1
Article Type:	Research
Date Submitted by the Author:	14-Sep-2017
Complete List of Authors:	Galson, Sophie; Duke University, Division of Emergency Medicine, Department of Surgery; Duke Global Health Institute<br>Staton, Catherine; Duke University, Division of Emergency Medicine, Department of Surgery; Duke Global Health Institute<br>Karia, Frank; Kilimanjaro Christian Medical University College<br>Kilonzo, Kajiru; Kilimanjaro Christian Medical University College, Medicine<br>Lunyera, Joseph; Duke University, Division of General Internal Medicine, Department of Medicine<br>Patel, Uptal; Duke University,<br>Hertz, Julian; Duke University, Division of Emergency Medicine, Department of Surgery; Duke Global Health Institute<br>Stanifer, John; Duke University, Division of Nephrology, Department of Medicine; Duke Global Health Institute
**Primary Subject Heading**:	Global health
Secondary Subject Heading:	Cardiovascular medicine, Qualitative research, Public health, Epidemiology
Keywords:	Hypertension < CARDIOLOGY, QUALITATIVE RESEARCH, health disparities, non-communicable diseases

SCHOLARONE™
Manuscripts

EPIDEMIOLOGY OF HYPERTENSION IN NORTHERN TANZANIA: A COMMUNITY-BASED MIXED-METHODS STUDY

CORRESPONDING AUTHOR:
Sophie W Galson, MD
310 Trent Drive, Duke Box 90518
Durham, NC 27708
Sophie.Galson@duke.edu
(301)-502-7718

AUTHORS:
Sophie W Galson, MD
Division of Emergency Medicine
Department of Surgery, Duke University; Durham, NC United States
Duke Global Health Institute

Catherine A Staton, MD MSc
Division of Emergency Medicine
Department of Surgery, Duke University; Durham, NC United States
Division of Global Neurosurgery and Neuroscience, Department of Neurosurgery
Duke Global Health Institute

Francis Karia, MBA, MSc-PH
Kilimanjaro Christian Medical College; Moshi, Tanzania

Kajiru Kilonzo, MD, MMed
Department of Medicine, Kilimanjaro Christian Medical Center; Moshi, Tanzania

Joseph Lunyera, MMBS
Division of General Internal Medicine
Department of Medicine, Duke University; Durham, NC United States

Uptal Patel, MD
Duke Clinical Research Institute
Department of Medicine, Duke University; Durham, NC United States

Julian T Hertz, MD
Division of Emergency Medicine
Department of Surgery, Duke University; Durham, NC United States
Duke Global Health Institute

John W Stanifer, MD, MSc
Division of Nephrology
Department of Medicine, Duke University; Durham, NC United States
Duke Global Health Institute

WORD COUNT: 4,545

For peer review only

ABSTRACT

Introduction: Sub-Saharan Africa (SSA) is particularly vulnerable to the growing global burden of hypertension but epidemiological studies are limited and barriers to optimal management are poorly understood. Therefore, we undertook a community-based mixed-methods study in Tanzania to investigate the epidemiology of hypertension and barriers to care.

Methods: In northern Tanzania, between December 2013 and June 2015, we conducted a mixed-method study, including a cross-sectional household epidemiological survey and qualitative sessions of focus groups and in-depth interviews. For the survey, we assessed for hypertension, defined as a single blood pressure ≥ 160/100 mmHg or a two-time average of ≥ 140/90 mmHg or current use of anti-hypertensive medications. To investigate relationships with potential risk factors, we used adjusted generalized linear models. Uncontrolled hypertension was defined as a two-time average measurement of ≥ 160/100 mmHg irrespective of treatment status. Hypertension awareness was defined a self-reported disease history in a participant with confirmed hypertension. To explore barriers to care, we identified emerging themes using an inductive approach within the Framework method.

Results: We enrolled 481 adults (median age 45) from 346 households, including 123 men (25.6%) and 358 women (74.4%). Overall, the prevalence of hypertension was 28.0% (95% CI 19.4-38.7), which was independently associated with age >60 years (prevalence risk ratio [PRR] 4.68; 95% CI 2.25-9.74) and alcohol use (PRR 1.72 (95% CI 1.15-2.58)). Traditional medicine use was inversely associated with hypertension (PRR 0.37 (0.26, 0.54)). Nearly half (48.3%) of the participants were aware of their disease, but almost all (95.3%) had uncontrolled hypertension. In the qualitative sessions, we identified barriers to optimal care, including poor point-of-care communication; poor understanding of hypertension; and structural barriers such as long wait-times and under-trained providers.

Conclusions: In northern Tanzania, the burden of hypertensive disease is substantial and optimal hypertension control is rare. Trans-disciplinary strategies sensitive to local practices should be explored to facilitate early diagnosis and sustained care delivery.

Keywords: Hypertension, Qualitative research, Health Disparities, Non-communicable diseases

For peer review only

STRENGTHS AND LIMITATIONS OF THIS STUDY

- Rigorous study design based on random, community-based sampling.
- The mixed-methods approach allowed for triangulation from multiple data sources with reproducible methods.
- Barriers to optimal hypertension care were explored using qualitative studies with key informants from both biomedicine and traditional medicine practices.
- As this was a cross-sectional study, causal inferences cannot be drawn.
- Our role as biomedical practitioners may have limited our ability to interpret results (researcher bias) about differences in disease understanding, and our inferences are derived from a biomedical perspective.

INTRODUCTION

Non-communicable diseases (NCDs), including hypertension, are a global epidemic disproportionately affecting health outcomes in low-and middle-income countries (LMICs) [1-3]. In sub-Saharan Africa (SSA) alone, more than 125 million people are expected to have hypertension by the year 2025 [4, 5]. Hypertensive-related complications are currently one of the leading causes of morbidity in SSA, and by 2030 hypertension and other NCDs are projected to surpass communicable diseases as the top cause of mortality [2, 3, 5]. Despite the overwhelming burden, SSA is mostly unprepared to address this impending public health crisis [6, 7].

Previously-identified barriers to addressing hypertension in SSA include under-recognition, under-treatment, and a limited understanding of its epidemiology [8, 9]. In particular, the lack of reliable health statistics and a paucity of community-based epidemiological data limit the ability for detection, surveillance, and creation of public health strategies for prevention and treatment [7, 10]. In northern Tanzania, for example, only 10-20% of patients with previously detected hypertension are receiving treatment, and only 16% of those on treatment were adequately controlled [11, 12].

Differences in hypertension care are related to several factors beyond healthcare access alone, including limited health literacy, cultural and social barriers, and heuristically different health belief models [13]. As such, understanding the epidemiology of hypertension as well as the social and community barriers to optimal care is critical for developing prevention and treatment strategies; however, few such studies have been conducted. Therefore, as part of the Comprehensive Kidney Disease Assessment for Risk Factors, Epidemiology, Knowledge, and Attitudes (CKD-AFRiKA) study, we conducted a mixed-methods study in order to characterize the community-based epidemiology of hypertension and barriers to optimal care through exploration of patient- and community-centered perspectives [14, 15].

METHODS

Ethics Statement

The study protocol was approved by Duke University Institutional Review Board (#Pro00040784), the Kilimanjaro Christian Medical College (KCMC) Ethics Committee (EC#502), and the National Institute for Medical Research

(NIMR) in Tanzania. Written informed consent (by signature or thumbprint) was obtained from all participants, and all participants with abnormal findings received counseling, educational pamphlets, and reimbursement with referral for follow-up. All participants were reimbursed between 1,500 and 12,000 Tanzanian Shillings (approximately 0.75 - 5.00 United States Dollars) depending upon their distance of travel.

Study Setting

We conducted a mixed methods study between December 2013 and June 2015 in the Kilimanjaro Region of Tanzania. The adult regional population is greater than 900,000 people, and it has a female majority (58%)[16]. Almost 35% of the adult population lives in an urban setting, which is comparable to national estimates, and the HIV prevalence is 3-5% [16, 17]. The unemployment rate is 19%, and most people have only a primary education (77%) [18]. The median age, average household size, and occupation distribution are similar to national estimates [16-18]. The largest ethnic group is the Chagga tribe, and Swahili is the major language [16]. The region comprises seven districts; our study was conducted in the Moshi Urban and Moshi Rural districts, which were selected based on their representative populations and proximity to our research infrastructure [16].

Quantitative Sampling and Data Collection

Detailed sampling methods for the CKD-AFRiKA Study have been previously reported [14, 15, 19]. In brief, a three-stage cluster probability sampling method, stratified by urban and rural setting, was used to randomly select neighborhoods based on probability proportional to size. Within each selected neighborhood, a cluster site was determined using geographic points randomly generated by Arc Global Information Systems (ArcGIS), v10.2.2 (Environmental Systems Research Institute, Redlands, CA). From the cluster site, households were then randomly chosen based on both a coin-flip and die-rolling technique according to our established protocol [14].

All non-pregnant, community-dwelling adults (age ≥ 18 years old) from the selected households were recruited into the study. The sample size was designed to estimate the prevalence of chronic kidney disease with a precision of 5% when accounting for the cluster-design effect. To reduce non-response rates, a minimum of two additional visits were attempted during off-hours (evenings and weekends) as well as multiple phone calls using mobile phone numbers.

All data were collected using trained, local surveyors. Each participant completed a demographic and medical history survey, which included self-reported history of diabetes, hypertension, HIV, kidney disease, and heart disease (coronary, structural, or heart failure). If participants were receiving biomedical treatment in the form of medical therapy, specific drug information was collected. Women additionally gave a self-reported history for pregnancy or menstruation. Awareness was defined as giving a self-reported history of hypertension and subsequently testing positive for hypertension in our screening process.

Anthropometric data (including height, weight, and body mass index) were recorded for each participant. Normal weight was defined as a BMI of 20 to 24.9 kg/m^2. Overweight was defined as a BMI ≥25 kg/m^2 and obesity was defined as a BMI ≥30 kg/m^2. We measured blood pressure using the automated Omron HEM-712 sphygmomanometer (Omron Healthcare, Inc.; Bannockburn, IL) that has an adjustable cuff size. The machine was calibrated monthly during data collection. All participants were seated in an erect position with feet flat on the floor for a minimum of five minutes before measurements. Two measurements separated by > 5 minutes were then performed. Hypertension was defined as a single blood pressure measurement of greater than 160/100 mmHg, a two-time average measurement of greater than 140/90 mmHg, or current self-reported use of anti-hypertensive medications. Uncontrolled hypertension was defined as a two-time average measurement

of greater than 160/100 mmHg irrespective of treatment status. Tobacco and alcohol use were defined as self-reported current ongoing use, former use, or never used.

Qualitative Data Collection

To explore patient- and community-centered perspectives related to barriers in optimal hypertension care, we conducted focus group discussions (FGDs) and in-depth interviews in a central, easily accessible location. These sessions have been described previously [20]. In brief, we conducted FGDs and in-depth interviews with key informants from the community including well-adults from the general population, chronically-ill adults receiving care at the hospital medicine clinics, adults receiving care from traditional healers, adults purchasing traditional medicines from herbal vendors, traditional healers, herbal vendors, and medical doctors. Purposive sampling was used to recruit the key informants. We targeted men and women of all ages from urban and rural settings with different education levels and ethnicities. FGDs were held in a rented office space in Moshi Urban that was well-known and easily accessible to local residents and ensured privacy. Each FGD lasted between four and six hours including breaks. In-depth interviews were conducted at the same office space with the exception of the traditional healers and herbal vendors who were interviewed at their places of work; these sessions lasted one to two hours. All sessions were semi-structured, open-ended, and probing. The discussion guide was initially written in English and then translated to Swahili by an independent team. All sessions were moderated by a native, local member of our team (FK). All sessions were audio-recorded, and two note-takers transcribed and independently translated each session. A moderator then reviewed the transcripts to ensure accuracy. Debriefings were held after each session, and team meetings were again held following translation.

Quantitative Data Analysis

The mean and standard deviation (SD) or median and inter-quartile ranges (IQR) were reported for continuous variables. Prevalence estimates were sample-balanced using age- and gender-weights based on the 2012 urban and rural district-level census data. We used a Chi-squared test or Fisher's exact test to compare differences between groups. All p-values are two-sided at a 0.05 significance level. Quantitative data were analyzed using STATAv.14 (STATA Corp., College Station, TX).

A secondary aim of the analysis was to explore associations between hypertension and potential risk factors related to lifestyle. Crude and adjusted prevalence risk ratios (PRR) were estimated using generalized linear models with a log link, and we used Taylor Series linearization to account for the design effect on variance due to cluster sampling. Separate uni-variable and multi-variable models were fitted to hypertension status for each lifestyle-related variable including alcohol use, tobacco use, traditional medicine use, living in an urban environment, and overweight/obesity status. Models were adjusted for confounding factors potentially associated with hypertension and each potential risk factor, including age, gender, and ethnicity. We did not include education or occupation in our models due to *a priori* assumptions about their potential upstream causal association with lifestyle-related risk factors.

All quantitative data were collected on paper and then electronically entered into and managed using REDCap electronic data capture tools hosted at Duke University [21]. All data were verified after electronic data entry by an independent reviewer to ensure accuracy.

Qualitative Data Analysis

We conducted a thematic analysis of the qualitative data by applying an inductive approach to the framework method [22]. The approach was based, in part, on our previously-developed model which explored

For peer review only

determinants of traditional medicine use and biomedical healthcare utilization among individuals in Kilimanjaro with NCDs, including hypertension [20]. After data reduction, we performed open-coding of all transcripts. We used a 'cultural insider' (emic) and a 'cultural outsider' (etic) to independently code the data. The cultural insider was a native researcher living in the region (FK) and the cultural outsider was a researcher foreign to the region (JWS). Comparisons were made between each code set and areas of disagreement were discussed and resolved by revisiting the data. This approach allowed us to explore concepts that otherwise may have been overlooked or misinterpreted by either researcher individually. The qualitative coding, analytic memos, and corresponding matrices were stored and analyzed using NViVOv.10.0 (QRS International Pty Ltd, Melbourne, Australia). The codes were grouped together into categories, and we used a coding index to formulate connections and explore relationships.

RESULTS

Table 1. Baseline Characteristics for the Quantitative Study

Variable (n, %)	Total (n=481)	Normotensive (n=332)	Hypertensive (n=149)	p-value
**Gender**				0.12
Male	123 (25.6%)	78 (23.5%)	45 (30.2%)	
Female	358 (74.4%)	254 (76.5%)	104 (69.8%)	
**Age**				<0.01
18-39 years old	172 (35.8%)	152 (45.8%)	20 (13.4%)	
40-59 years old	191 (39.7%)	132 (39.8%)	59 (39.6%)	
60+ years old	118 (24.5%)	48 (14.5%)	70 (47.0%)	
**Ethnicity**				0.40
Chagga	288 (59.9%)	193 (58.1%)	95 (63.7%)	
Pare	66 (13.7%)	51 (15.4%)	15 (10.1%)	
Sambaa	27 (5.6%)	20 (6.0%)	7 (4.7%)	
Other[§]	100 (20.8%)	68 (20.5%)	32 (21.5%)	
**Education**				<0.01
None	31 (6.4%)	11 (3.31%)	20 (13.4%)	
Primary	349 (72.6%)	246 (74.1%)	103 (69.1%)	
Secondary	74 (15.4%)	54 (16.3%)	20 (13.4%)	
Post-Secondary	27 (5.6%)	21 (6.3%)	6 (4.03%)	
**Occupation**				<0.01
Unemployed[#]	74 (15.4%)	55 (16.6%)	19 (12.8%)	
Farmer/Wage Earner	199 (41.4%)	135 (40.7%)	64 (43.0%)	
Small Business/Vendors	158 (32.8%)	121 (36.5%)	37 (24.8%)	
Professional[†]	50 (10.4%)	21 (6.3%)	29 (19.5%)	
**Lifestyle Practices**				
Ongoing tobacco use	50 (10.4%)	34 (10.2%)	16 (10.7%)	0.87
Ongoing alcohol use	198 (41.2%)	121 (36.4%)	77 (51.7%)	0.02
Traditional medicine use	272 (56.6%)	196 (59.0%)	76 (51.0%)	0.10
**Self-Reported Medical History**				
Diabetes	61 (12.7%)	29 (8.7%)	32 (21.5%)	<0.01
Hypertension	134 (28.0%)	62 (18.8%)	72 (48.3%)	<0.01
Stroke	8 (1.7%)	2 (0.6%)	6 (4.0%)	0.01
Heart Disease[*]	18 (3.7%)	7 (2.1%)	7 (4.7%)	0.08
Kidney Disease	14 (2.9%)	10 (3.0%)	4 (2.7%)	0.84

Table 2. Baseline Characteristics for the Qualitative Study

	FGD1	FGD2	FGD3	FGD4	FGD5	In-Depth Interviews
**Study Population**	Clinic Patients	General Population	Clinic Patients	General Population	Medical Doctors	Patients from Healers and Vendors
**Participants (N)**	15	12	16	12	4	11
**Gender**						
Male	0 (0%)	0(0%)	16(100%)	12(100%)	2(50%)	5(45%)
Female	15(100%)	12(100%)	0(0%)	0(0%)	2(50%)	6(55%)
Age range (years)	25-61	26-65	18-70	18-74	30-36	19-60
**Ethnicity**						
Chagga	11 (73%)	9 (75%)	11 (69%)	4 (33%)	2 (50%)	2 (18%)
Pare	2 (13%)	2 (17%)	2 (13%)	5 (42%)	0	0
Maasai	0	0	0	0	0	4 (36%)
Sambaa	1 (7%)	1 (8%)	1 (6%)	0	0	3 (27%)
Other*	1 (7%)	0	2 (13%)	3 (25%)	2 (50%)	2 (18%)
**Education**						
None	0	0	0	0	0	2 (18%)
Primary	11 (73%)	10 (83%)	10 (63%)	3 (25%)	0	4 (36%)
Secondary	3 (20%)	2 (17%)	5 (31%)	6 (50%)	0	1 (9%)
University	1 (7%)	0	1 (6%)	3 (25%)	4 (100%)	4 (36%)
**Occupation**						
Unemployed[#]	2 (13%)	4 (33%)	0	1 (8%)	0	3 (27%)
Student	0	0	4 (25%)	5 (42%)	0	0
Farmer/Wage Earner	4 (27%)	3 (25%)	8 (50%)	3(25%)	0	5 (45%)
Small Business	3 (20%)	2 (17%)	3 (19%)	2 (17%)	0	1 (9%)
Professional[†]	4 (27%)	3 (25%)	1 (6%)	1 (8%)	4(100%)	2 (18%)
**Religion**						
Roman Catholic	5 (33%)	5 (42%)	8 (50%)	1 (8%)	3 (75%)	7 (64%)
Lutheran	6 (40%)	4 (33%)	4 (25%)	2 (17%)	0	1 (9%)
Christian Evangelical	1 (7%)	1 (8%)	2 (13%)	5 (42%)	1 (25%)	1 (9%)
Christian (Other)	2 (13%)	0	0	0	0	0
Islam	1 (7%)	2 (17%)	2 (13%)	4 (33%)	0	2 (18%)
**Residence**						
Urban	9 (60%)	11 (92%)	10 (83%)	12 (100%)	4 (100%)	9 (82%)
Rural	6 (40%)	1 (8%)	2 (17%)	0 (0%)	0 (0%)	2 (18%)

Study Populations

We enrolled 481 adults into the quantitative study (**Table 1**). The median age was 45.0 years (IQR 35–59). The majority of participants were women (n = 358; 74%), lived in an urban location (n = 370; 77%), ethnically Chagga (n = 288; 60%), and only had a primary school education (n = 349; 73%). The most common occupation among participants was farming or daily wage work (n =199; 41%). Many participants reported ongoing use of alcohol (n=198; 41%) and traditional medicine use over the previous year (n=272; 57%), with the most commonly reported frequency of traditional medicine use at 1–5 times (31.0 %) per year. Among participates currently using prescribed biomedicines (n=70), the proportion of participants reporting traditional medicine use was more substantial, with 69% (n=48) reporting traditional medicine use over the previous year.

The household non-response rate was 15.0% and the individual non-response rate was 20.6%. Compared with the regional population [16], men (p<0.001) and young adults 18–39 years old (p = 0.001) were more likely to be non-responders in our study, and the proportion of participants with a secondary or post-secondary education

(22%) was higher than the regional average (15%) (p = 0.02). We observed no significant differences in occupation between the responders and non-responders (p = 0.64).

In the qualitative study, we conducted five FGDs and 11 in-depth interviews (**Table 2**). FGDs and in-depth interviews included even numbers of men (n= 35; 50%), and women (n=35; 50%) and had an age range of 18 to 74 years. Most participants were of the Chagga ethnic group (n=37; 53%), and were Roman Catholic (n=29; 41%), but Islamic (n=11; 16%), Lutheran (n=17; 24%), and Christian Evangelical (n=11; 16%) were also represented as well as thirteen different tribal ethnicities. Education levels varied from none (n=2; 3%) to university level (n=13; 19%), but the majority had only completed a primary education (n=38; 54%). Most participants were from urban residences (n=55; 79%).

Burden of Hypertension

The prevalence of hypertension was 28% (95% CI 19.4-38.7). The design effect of the cluster sampling was 2.34, with a neighborhood-level ICC coefficient of 0.075. Mean systolic blood pressure was 129.5 mmHg (SD 24.3), mean diastolic blood pressure was 77.6 mmHg (SD 12.2), and the median age was 58 years (IQR 45-65). The prevalence of hypertension was 59.3% (95% CI 50.1-67.9) in participants ≥ 60 years old. Comparatively, prevalence was 30.9% (95% CI 24.7-37.9) in the 40-59 age group and 11.6% (95% CI 7.6-17.4) in the under 40-age group. The median BMI of individuals with hypertension was 27.4 kg/m^2 (IQR 24-30). Participants with hypertension were more likely to be men, older, report ongoing alcohol use, live in an urban environment, have less education, and be employed as professionals (p<0.05 for all) (**Table 1**). The proportion of reported traditional medicine use among participants with hypertension was 39.3% (95% CI 30.3-49.1%), and the most common reasons reported for using traditional medicines were to treat daily symptomatic ailments (45%) and for treatment of chronic diseases (10%), including hypertension (**Figure 1**).

Crude and adjusted prevalence risk ratios for the relation between lifestyle-related factors and hypertension are reported in **Table 3**. In crude models, traditional medicine use was inversely associated with hypertension prevalence (PRR 0.60; 95% CI 0.41-0.87), and alcohol use was significantly associated with higher prevalence of hypertension (PRR 2.29; 95% 1.26-4.15). These associations remained significant even after adjustment for age, gender, and ethnicity, with a PRR 0.37 (95% CI 0.26-0.54) and PRR 1.72 (95% CI 1.15-2.58) for traditional medicine use and alcohol use, respectively. We did not observe an association between hypertension prevalence and obesity, urban residence, or tobacco use (p>0.05 for all).

Table 3. Associations between lifestyle factors and hypertension; CKD-AFRIKA, 2015.

Variables	Prevalence Risk Ratios (95% CI)	
	Unadjusted	Adjusted*
Ongoing Tobacco Use	1.25 (0.75, 2.10)	0.68 (0.41, 1.14)
Traditional Medicine Use	**0.60 (0.41, 0.87)**	**0.37 (0.26, 0.54)**
Ongoing Alcohol Use	**2.29 (1.26, 4.15)**	**1.72 (1.15, 2.58)**
Overweight/Obese	1.00 (0.57, 1.74)	1.28 (0.84, 1.97)
Urban Residence	0.58 (0.34, 1.00)	0.85 (0.59, 1.22)

*Adjusted for age, gender, and ethnicity
BOLD: Significant at the 5% level

Barriers to Optimal Care

For peer review only

Despite the high disease burden, only half of participants with elevated blood pressure (48%) were aware of having hypertension. Few (23%) reported taking biomedicines for hypertension, and 12% reported taking both biomedicines and traditional medicines. Almost all participants (95%) had uncontrolled hypertension. A major theme that emerged as an important barrier for awareness and disease self-management was a difference in individual chronic disease understanding. Most notably, we identified quality or perceived quality of the biomedical healthcare delivery, disease expression, chronicity of disease, and traditional health belief models as important contributors to the observed differences in chronic disease understanding, which itself contributed to unrealistic expectations of cure, perceived treatment failures, and medical non-compliance (**Figure 2**).

Participants related structural issues and point-of-care communication issues as key barriers to optimal biomedical healthcare delivery. Structural issues in the delivery of biomedical healthcare, including long wait times, under-staffing, lack of experience by healthcare providers, and medication costs, were particularly concerning, and together with poor point-of-care communication between patient and provider, appeared to substantially contribute to differences in chronic disease understanding through unrealistic expectations of cure expressed as concern over ineffective or inappropriate therapies:

> *MDs have a lot of patients to take care of so they lack enough time to give explanations. They hurry so that they can save as many patients as possible.*
>
> *I attended the hospital and treatment was begun but with no success for a long time; instead other parts in my body began to swell. At that time I decided to discharge myself from the hospital, and my grandmother gave me local herbals which cured me.*

For many participants, differences in disease understanding were also closely related to the disease expression or the symptom complex of disease. For chronic, generally asymptomatic diseases such as hypertension, this led to unrealistic expectations of cure, greater perceptions of treatment failure by biomedicines, and increased medical non-compliance:

> *You know you have a disease because the body always has symptoms.*
>
> *You know you are healed as you do not have to attend the hospital anymore because your symptoms have disappeared.*

For chronic diseases such as hypertension, chronicity or duration of disease was an especially salient topic closely related to differences in disease understanding. Chronic diseases were understood to be diseases that have either been untreated or undertreated, and even infectious diseases were viewed as chronic when left untreated:

> *Anything that stays in the human body for a long time without being cured, like amoebae and bilharzias* [schistosomiasis] *is a chronic disease.*

For peer review only

From the biomedical perspective, this different understanding of chronicity led to many challenges in achieving optimal care for patients with hypertension, particularly with respect to medication compliance, expectations of cure, and perceived treatment failure. As biomedical doctors explained:

> *Some patients with hypertension, after their blood pressure is controlled, they then believe they are cured after 2 or 3 months. They go to follow-up and see that their blood pressure normal; therefore, they assume they are cured and stop their medications. The chronicity of the disease they do not understand well.*

> *…the people keep on seeking a 'cure' for something that is a chronic disease.*

Finally, traditional health belief models were also closely associated with differences in disease understanding. Even for chronic diseases such as hypertension, participants expressed the importance of traditional medicines, and community elders and family members were considered important sources of healthcare knowledge.

> *My family and I prefer not to go to hospitals. My grandparents taught us a lot (especially about plant roots) about healing and curing… my father still will not use any hospital medicines.*

> *…most of the chronic diseases are cured by traditional medicines.*

Discussion

In a community-based setting in northern Tanzania, we found an alarmingly high burden of hypertension. The prevalence observed was 10-20% higher than similar regions in SSA and comparable with several upper and middle-income countries, including South Africa, Brazil, and China [2, 7, 9, 12, 23-25]. Despite the high burden of hypertension, awareness was low and few had achieved optimal blood pressure control, which may be explained by observed gaps in communication, quality of healthcare delivery, and traditional medicine health belief models. In particular, we identified differences in disease understanding as they relate to a disease expression through symptom complexes, disease chronicity, and traditional health beliefs as potentially important barriers for achieving optimal hypertension care.

As epidemiological transitions reshape the region, Tanzania is at great risk for an explosive growth in the burden of NCDs, including hypertension [7, 26, 27]. The rapid pace of urbanization and economic growth are accelerating the rate of this transition; thus, as evidenced by the high prevalence we are already observing, there is an urgent need for action [24]. Aggressive efforts should be made to diagnose and capture hypertensive patients at every single interaction within health systems. Considering the low rates of awareness in our study, all settings for diagnosis and delivery of healthcare should be explored including community centers and traditional medicine providers. Community-centered care models may be beneficial to reduce risk factors, improve treatment adherence, and have been successful previously in SSA [6, 28]. Given the important role of traditional medicine practices and prior willingness to refer patients to biomedical facilities for diagnostic testing [29], partnership with traditional medicine providers should be considered to assist with risk factor reduction and care coordination, including early referral [29, 30].

Additionally, emergency department (ED)-based screening has also been successful at capturing undiagnosed/uncontrolled hypertension cases and linking patients to care in high-income countries; yet ED-

For peer review only

based care models for hypertension have not been widely explored in SSA [31-33]. For example, ED-based interventions to screen for hypertension and modifiable risk factors as the first step of a care pathway may prove to be highly effective in hypertension care. Beyond diagnosis, careful consideration of the local environment and barriers to care will be necessary to create successful educational programs and sustain hypertension control. Educational interventions should focus on the concepts of chronicity and disease expression, and incorporate traditional health beliefs. Targeted and culturally-tailored engagement with patients may prevent poor hypertension outcomes through improved self-management, particularly with medication compliance, expectations of cure, and perceived treatment success [34].

The application of locally-tailored programs will also require a comprehensive understanding of traditional medicine practices, particularly as they intersect with biomedical concepts of health. We found traditional medicines use was substantially high but associated with a lower prevalence of hypertension, and more work is needed to understand whether this could indicate a protective effect or alternatively, a form of selection bias, reporting bias, or unmeasured confounding. We also identified high concurrent use of traditional medicines and biomedicines, which further stresses the importance of culturally competent interventions when addressing hypertension. Previous research by our group in the region found that traditional medicines are used by 50%-70% of the general population, consistent with other reports across Tanzania [20, 35, 36]. Specifically, in the current study, among those with hypertension reporting the ongoing use of biomedicine, over 60% of participants were concurrently using traditional medicines, spanning all incomes, education levels and residential settings. Although we previously identified 168 separate plant-based traditional medicines used in this region, including two used specifically for treatment of hypertension (Lemongrass (*Cymbopogon citrullus)* and Erabel *(scientific name unknown),* further research is greatly needed to classify traditional plant-based-medicines, including their mechanisms of action, side effect profiles, and potential protective effects.

In contrast to traditional medicines, we found alcohol to be positively associated with hypertension in this population, consistent with trends throughout SSA [37-39]. This region of Tanzania has a particularly high prevalence of alcohol use due to widespread cultural acceptability and home-brewing culture [40]. Given the high burden of alcohol, practitioners in all locations should be cognizant of this risk factor during both hypertension screening and treatment. Conversely, hypertension prevalence was not associated with obesity, urban residence or tobacco use, all well-established risk factors in high-income countries [38, 41]. While prevalence was high in both rural and urban settings, it is unclear what factors may contribute to the chronic disease burdens across SSA and the drivers of hypertension may be different in urban vs. rural settings (e.g. dietary changes, environmental exposures, or differential access to care) [42, 43]. As with other chronic diseases, the link between obesity and hypertension is also not well understood in sub-Saharan Africa [44], and it is currently unclear to what extent genetic, lifestyle, and environmental interact to drive hypertension disparities [26, 45]. As such, future work investigating the determinants and risk factors for hypertension in this setting is urgently needed.

Our study was unique in exploring hypertension in the Kilimanjaro area by utilizing a rigorous randomly-sampled, household-level survey as part of a mixed-method design that also included qualitative sessions with key informants. We explored latent themes and social/community context for treatment failure, and by leveraging this thematic analysis we were able to identify targetable barriers to optimal hypertension diagnosis, treatment, and control. Nonetheless, we noted potential limitations to our study. First, selection bias from non-response may be present. To address any non-response bias that may have arisen from differences between the respondents and non-respondents, we used sample-balanced weights for age and gender and explored differences in occupation and education level between the two groups. In regards to internal validity, we only measured blood pressure at one setting; however, two measurements separated by > 5 minutes were

For peer review only

performed and previous studies have shown that sustained elevated blood pressure in one setting may be sensitive to establish a diagnosis of hypertension [32]. Misclassification of disease around the cutoff points for hypertension may also be present although we expect this misclassification to be non-differential. Medical history was also determined by self-report for several conditions, which may be less accurate in this setting with low awareness. Also, alcohol and tobacco use were not quantified and future research would be strengthened by quantifying tobacco and alcohol use with validated instruments [46]. Additionally, as this was a cross-sectional study, causal inferences cannot be drawn and associations may be influenced by confounding from unmeasured variables. Our role as biomedical practitioners may have limited our ability to interpret results (researcher bias) about differences in disease understanding, and our inferences are derived from a biomedical perspective. Finally, although we used insider-outsider coding, local non-medical surveyors, and local moderators for qualitative data collection, reporting bias may still be present.

In conclusion, in a community-based study of adults from the Kilimanjaro region of northern Tanzania we observed a high prevalence of hypertension, most of which was uncontrolled. Alcohol use may be an important risk factor for hypertension, and we identified several emerging cultural and social themes as barriers to optimal hypertension care, including most notably difference in disease understanding related to quality of healthcare delivery, chronicity of disease, disease expression, and traditional belief models. Hypertension care-models will need to leverage all existing resources from the ED, to community centers, to traditional healers in order to address the growing burden of hypertension in the region, and future studies are needed to develop targeted, culturally-tailored interventions designed to improve hypertension disease understanding.

ACKNOWLEDGMENTS

We would like to thank Professors G. Ralph Corey and John Bartlett, and all the staff of the KCMC-Duke Collaboration in Moshi, Tanzania for all of their efforts on the Comprehensive Kidney Disease Assessment For Risk factors, epidemiology, Knowledge, and Attitudes (CKD AFRiKA) Study. We give a special thanks to Carol Sangawe, Cynthia Asiyo, Nicola West, and Edith Aloyce Macha for their integral role implementing the study and Jeffrey Hawley and Audrey Brown at the Duke Office of Clinical Research for their help in data management. We are also grateful to Estomih Mduma and his team for their help with Swahili translation needs.

CONTRIBUTION

JS and UP developed the concept of the project. JS and FK conducted data collection. SG, JS, and JL contributed to writing the manuscript. JS and JL were responsible for the analysis plan and data analysis. CS, JS, SG, JL, KK, JH, UP, and FK were responsible for the final editing and all authors approved the final manuscript.

COMPETING INTERESTS

All authors have completed the ICMJE uniform disclosure form at www.icmje.org/coi_disclosure.pdf and declare: no support from any organization for the submitted work; no financial relationships with any organizations that might have an interest in the submitted work in the previous three years; no other relationships or activities that could appear to have influenced the submitted work.

FUNDING

This study was supported by an NIH Research Training Grant (#R25 TW009337) funded by the Fogarty International Center and the National Institute of Mental Health; and a Research and Prevention Grant funded

For peer review only

by the International Society of Nephrology Global Outreach Committee. Dr. Staton would like to acknowledge salary support funding from the Fogarty International Center (Staton, K01 TW010000-01A1).

DATA SHARING

We have concerns about the ethics of openly releasing the entire dataset to the public as the structure of the dataset would result in loss of participant anonymity. However, we will ensure that the dataset is openly available to researchers who contact us and meet confidentiality requirements (documentation of ethics training in conduct of human-subject research). They may contact Dr. John W Stanifer, DCRI 2400 Pratt St, Durham NC 27710 or john.stanifer@duke.edu

FIGURE LEGEND

Fig. 1 Reported reasons for using traditional medicines among participants with hypertension.

Fig. 2 Conceptual model describing the hypothesized relationship between disease understanding and hypertension outcomes.

For peer review only

REFERENCES

1. Abegunde DO, Mathers CD, Adam T, et al. The burden and costs of chronic diseases in low-income and middle-income countries. *Lancet (London, England)*. 2007;370(9603):1929-38.
2. WHO | A global brief on hypertension. *WHO*. 2013.
3. Institute for Health Metrics and Evaluation (IHME) 2015 [Available from: http://vizhub healthdata org/gbd-compare.
4. Pastakia SD, Ali SM, Kamano JH, et al. Screening for diabetes and hypertension in a rural low income setting in western Kenya utilizing home-based and community-based strategies. *Globalization and health*. 2013;9:21.
5. Whitworth JA. 2003 World Health Organization (WHO)/International Society of Hypertension (ISH) statement on management of hypertension. *Journal of hypertension*. 2003;21(11):1983-92.
6. Twagirumukiza M, Van Bortel LM. Management of hypertension at the community level in sub-Saharan Africa (SSA): towards a rational use of available resources. *Journal of human hypertension*. 2011;25(1):47-56.
7. Peck R, Mghamba J, Vanobberghen F, et al. Preparedness of Tanzanian health facilities for outpatient primary care of hypertension and diabetes: a cross-sectional survey. *Lancet Glob Health*. 2014;2(5):e285-92.
8. Naanyu V, Vedanthan R, Kamano JH, et al. Barriers Influencing Linkage to Hypertension Care in Kenya: Qualitative Analysis from the LARK Hypertension Study. *Journal of general internal medicine*. 2016;31(3):304-14.
9. Chow CK, Teo KK, Rangarajan S, et al. Prevalence, awareness, treatment, and control of hypertension in rural and urban communities in high-, middle-, and low-income countries. *Jama*. 2013;310(9):959-68.
10. Churchill LO. Epidemiology of ischaemic heart disease in sub-Saharan Africa. *Cardiovasc J Afr*. 2013;24(2):34-42.
11. Dewhurst MJ, Dewhurst F, Gray WK, et al. The high prevalence of hypertension in rural-dwelling Tanzanian older adults and the disparity between detection, treatment and control: a rule of sixths? *Journal of human hypertension*. 2012;27(6):374-80.

12. Mosha NR, Mahande M, Juma A, et al. Prevalence,awareness and factors associated with hypertension in North West Tanzania. *Global health action*. 2017;10(1):1321279.
13. Nnko S, Bukenya D, Kavishe BB, et al. Chronic Diseases in North-West Tanzania and Southern Uganda. Public Perceptions of Terminologies, Aetiologies, Symptoms and Preferred Management. *PloS one*. 2015;10(11):e0142194.
14. Stanifer JW, Maro V, Egger J, et al. The epidemiology of chronic kidney disease in Northern Tanzania: a population-based survey. *PloS one*. 2015;10(4):e0124506.
15. Stanifer JW, Egger JR, Turner EL, et al. Neighborhood clustering of non-communicable diseases: results from a community-based study in Northern Tanzania. *BMC Public Health*. 2016;16:226.
16. Central Census Office and National Bureau of Statistics. United Republic of Tanzania: 2012 Population and Housing Census 2013 [Available from: http://www.nbs.go.tz. .
17. Demographic and Health Surveys. Program. United Republic of Tanzania HIV/AIDS and Malaria Indicator Survey 2011-2012. 2013 [Available from: http://dhsprogram.com/pubs/pdf/AIS11/AIS11.pdf.
18. Development Committee. United Republic of Tanzania: Education Sector Performance Report, 2010-2011 2013 [Available from: http://www.ed-dpg.or.tz.
19. Stanifer JW, Cleland CR, Makuka GJ, et al. Prevalence, Risk Factors, and Complications of Diabetes in the Kilimanjaro Region: A Population-Based Study from Tanzania. *PloS one*. 2016;11(10):e0164428.
20. Stanifer JW, Patel UD, Karia F, et al. The determinants of traditional medicine use in Northern Tanzania: a mixed-methods study. *PloS one*. 2015;10(4):e0122638.
21. Harris PA, Taylor R, Thielke R, et al. Research electronic data capture (REDCap)--a metadata-driven methodology and workflow process for providing translational research informatics support. *Journal of biomedical informatics*. 2008;42(2):377-81.
22. Gale NK, Heath G, Cameron E, et al. Using the framework method for the analysis of qualitative data in multi-disciplinary health research. *BMC medical research methodology*. 2013;13:117.
23. Pastakia SD, Ali SM, Kamano JH, et al. Screening for diabetes and hypertension in a rural low income setting in western Kenya utilizing home-based and community-based strategies. Globalization and health. 92013. p. 21.
24. Twagirumukiza M, De Bacquer D, Kips JG, et al. Current and projected prevalence of arterial hypertension in sub-Saharan Africa by sex, age and habitat: an estimate from population studies. *Journal of hypertension*. 2011;29(7):1243-52.
25. Kearney PM, Whelton M, Reynolds K, et al. Worldwide prevalence of hypertension: a systematic review. *Journal of hypertension*. 2004;22(1):11-9.
26. Yikona J. Non-communicable disease in sub-Saharan Africa. *Lancet (London, England)*. 2001;357(9249):74.
27. Mufunda J, Chatora R, Ndambakuwa Y, et al. Emerging non-communicable disease epidemic in Africa: preventive measures from the WHO Regional Office for Africa. *Ethnicity & disease*. 2006;16(2):521-6.
28. Jeffries D. Risk in cardiovascular disease. Having so many different guidelines about reducing risk is confusing. *BMJ (Clinical research ed)*. 2000;321(7254):175.
29. Liwa AC, Smart LR, Frumkin A, et al. Traditional Herbal Medicine Use Among Hypertensive Patients in Sub-Saharan Africa: A Systematic Review. *Curr Hypertens Rep*. 2014;16(6):437.
30. Gessler MC, Msuya DE, Nkunya MH, et al. Traditional healers in Tanzania: sociocultural profile and three short portraits. *Journal of ethnopharmacology*. 1995;48(3):145-60.
31. Chernow SM, Iserson KV, Criss E. Use of the emergency department for hypertension screening: a prospective study. *Annals of emergency medicine*. 1987;16(2):180-2.
32. Ackerson HDB, Linda D, Lynn. Reproducibility of increased blood pressure during an emergency department or urgent care visit. *Annals of emergency medicine*. 2003;41(4):507-12.
33. Tan N, Taylor DM. Feasibility and outcomes of screening for cardiovascular risk factors in the emergency department. *Emergency medicine Australasia : EMA*. 2013;25(2):175-81.

For peer review only

34. Warsi A WP, LaValley MP, Avorn J, Solomon DH. Self-management education programs in chronic disease: a systematic review and methodological critique of the literature. *Arch Intern Med*. 2004:9-23;164(15):1641-9.
35. Kayombo EJ, Uiso FC, Mahunnah RL. Experience on healthcare utilization in seven administrative regions of Tanzania. *Journal of Ethnobiology and Ethnomedicine*. 2012;8(1):5.
36. Liwa AC, Smart LR, Frumkin A, et al. Traditional Herbal Medicine Use Among Hypertensive Patients in Sub-Saharan Africa: A Systematic Review. *Current Hypertension Reports*. 2014;16(6):437.
37. Xin X, He J, Frontini MG, et al. Effects of alcohol reduction on blood pressure: a meta-analysis of randomized controlled trials. *Hypertension*. 2001;38(5):1112-7.
38. He J, Bazzano LA. Effects of lifestyle modification on treatment and prevention of hypertension. *Current opinion in nephrology and hypertension*. 2000;9(3):267-71.
39. Nahimana MR, Nyandwi A, Muhimpundu MA, et al. A population-based national estimate of the prevalence and risk factors associated with hypertension in Rwanda: implications for prevention and control. *BMC Public Health*. 2017;18(1):2.
40. Mitsunaga T, Larsen U. Prevalence of and risk factors associated with alcohol abuse in Moshi, northern Tanzania. *Journal of biosocial science*. 2008;40(3):379-99.
41. Pereira M, Lunet N, Azevedo A, et al. Differences in prevalence, awareness, treatment and control of hypertension between developing and developed countries. *Journal of hypertension*. 2009;27(5):963-75.
42. Njelekela M, Sato T, Nara Y, et al. Nutritional variation and cardiovascular risk factors in Tanzania--rural-urban difference. *South African medical journal = Suid-Afrikaanse tydskrif vir geneeskunde*. 2003;93(4):295-9.
43. Damasceno A, Azevedo A, Silva-Matos C, et al. Hypertension prevalence, awareness, treatment, and control in mozambique: urban/rural gap during epidemiological transition. *Hypertension*. 2009;54(1):77-83.
44. Okosun IS, Rotimi CN, Forrester TE, et al. Predictive value of abdominal obesity cut-off points for hypertension in blacks from west African and Caribbean island nations. *International journal of obesity and related metabolic disorders : journal of the International Association for the Study of Obesity*. 2000;24(2):180-6.
45. Mathenge W, Foster A, Kuper H. Urbanization, ethnicity and cardiovascular risk in a population in transition in Nakuru, Kenya: a population-based survey. *BMC Public Health*. 2010;10:569.
46. Lundin A, Hallgren M, Balliu N, et al. The use of alcohol use disorders identification test (AUDIT) in detecting alcohol use disorder and risk drinking in the general population: validation of AUDIT using schedules for clinical assessment in neuropsychiatry. *Alcoholism, clinical and experimental research*. 2015;39(1):158-65.

For peer review only

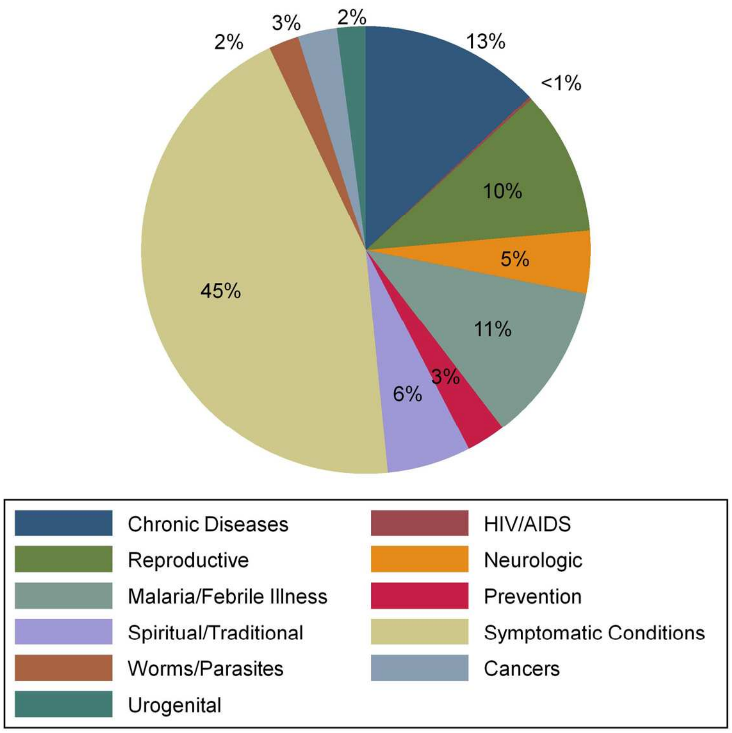

Figure 1. Reported reasons for using traditional medicines among participants with hypertension

86x64mm (600 x 600 DPI)

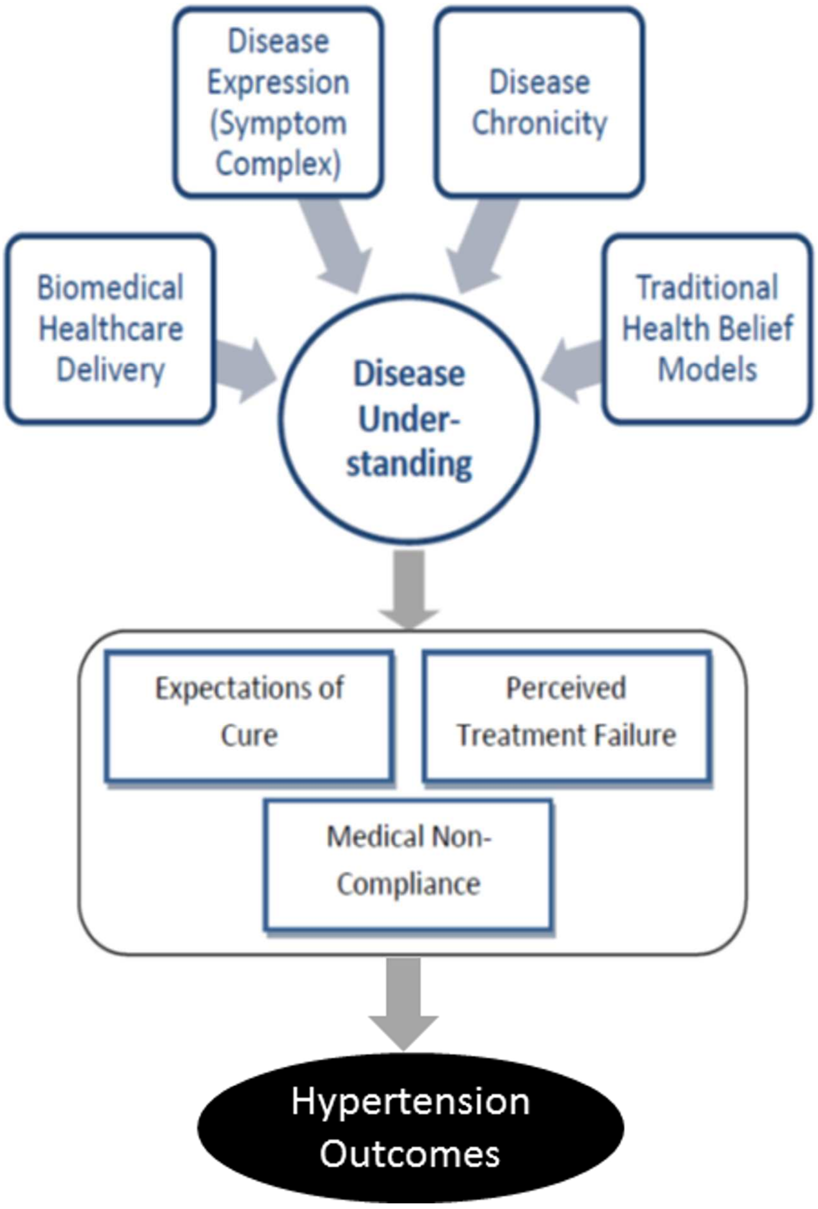

Figure 2. Conceptual model describing the hypothesized relationship between disease understanding and hypertension outcomes

189x279mm (300 x 300 DPI)

STROBE 2007 (v4) Statement—Checklist of items that should be included in reports of *cross-sectional studies*

Section/Topic	Item #	Recommendation	Reported on page #
**Title and abstract**	1	(*a*) Indicate the study's design with a commonly used term in the title or the abstract	1
		(*b*) Provide in the abstract an informative and balanced summary of what was done and what was found	2
**Introduction**			
Background/rationale	2	Explain the scientific background and rationale for the investigation being reported	3
Objectives	3	State specific objectives, including any prespecified hypotheses	3
**Methods**			
Study design	4	Present key elements of study design early in the paper	4
Setting	5	Describe the setting, locations, and relevant dates, including periods of recruitment, exposure, follow-up, and data collection	4
Participants	6	(*a*) Give the eligibility criteria, and the sources and methods of selection of participants	4
Variables	7	Clearly define all outcomes, exposures, predictors, potential confounders, and effect modifiers. Give diagnostic criteria, if applicable	4
Data sources/ measurement	8*	For each variable of interest, give sources of data and details of methods of assessment (measurement). Describe comparability of assessment methods if there is more than one group	4
Bias	9	Describe any efforts to address potential sources of bias	*4*
Study size	10	Explain how the study size was arrived at	4
Quantitative variables	11	Explain how quantitative variables were handled in the analyses. If applicable, describe which groupings were chosen and why	5
Statistical methods	12	(*a*) Describe all statistical methods, including those used to control for confounding	5
		(*b*) Describe any methods used to examine subgroups and interactions	5
		(*c*) Explain how missing data were addressed	5
		(*d*) If applicable, describe analytical methods taking account of sampling strategy	5
		(*e*) Describe any sensitivity analyses	NA
**Results**			

For peer review only

Participants	13*	(a) Report numbers of individuals at each stage of study—eg numbers potentially eligible, examined for eligibility, confirmed eligible, included in the study, completing follow-up, and analysed	4
		(b) Give reasons for non-participation at each stage	4
		(c) Consider use of a flow diagram	4 (reference)
Descriptive data	14*	(a) Give characteristics of study participants (eg demographic, clinical, social) and information on exposures and potential confounders	6
		(b) Indicate number of participants with missing data for each variable of interest	4
Outcome data	15*	Report numbers of outcome events or summary measures	6,7
Main results	16	(*a*) Give unadjusted estimates and, if applicable, confounder-adjusted estimates and their precision (eg, 95% confidence interval). Make clear which confounders were adjusted for and why they were included	6,7
		(*b*) Report category boundaries when continuous variables were categorized	4
		(*c*) If relevant, consider translating estimates of relative risk into absolute risk for a meaningful time period	NA
Other analyses	17	Report other analyses done—eg analyses of subgroups and interactions, and sensitivity analyses	8
**Discussion**			
Key results	18	Summarise key results with reference to study objectives	10
Limitations	19	Discuss limitations of the study, taking into account sources of potential bias or imprecision. Discuss both direction and magnitude of any potential bias	11
Interpretation	20	Give a cautious overall interpretation of results considering objectives, limitations, multiplicity of analyses, results from similar studies, and other relevant evidence	10
Generalisability	21	Discuss the generalisability (external validity) of the study results	11
**Other information**			
Funding	22	Give the source of funding and the role of the funders for the present study and, if applicable, for the original study on which the present article is based	11

*Give information separately for cases and controls in case-control studies and, if applicable, for exposed and unexposed groups in cohort and cross-sectional studies.

Note: An Explanation and Elaboration article discusses each checklist item and gives methodological background and published examples of transparent reporting. The STROBE checklist is best used in conjunction with this article (freely available on the Web sites of PLoS Medicine at http://www.plosmedicine.org/, Annals of Internal Medicine at http://www.annals.org/, and Epidemiology at http://www.epidem.com/). Information on the STROBE Initiative is available at www.strobe-statement.org.

For peer review only

BMJ Open

EPIDEMIOLOGY OF HYPERTENSION IN NORTHERN TANZANIA: A COMMUNITY-BASED MIXED-METHODS STUDY

Journal:	*BMJ Open*
Manuscript ID	bmjopen-2017-018829.R2
Article Type:	Research
Date Submitted by the Author:	13-Oct-2017
Complete List of Authors:	Galson, Sophie; Duke University, Division of Emergency Medicine, Department of Surgery; Duke Global Health Institute<br>Staton, C; Duke University, Division of Emergency Medicine, Department of Surgery; Duke Global Health Institute<br>Karia, Frank; Kilimanjaro Christian Medical University College<br>Kilonzo, Kajiru; Kilimanjaro Christian Medical University College, Medicine<br>Lunyera, Joseph; Duke University, Division of General Internal Medicine, Department of Medicine<br>Patel, UD; Duke University,<br>Hertz, Julian; Duke University, Division of Emergency Medicine, Department of Surgery; Duke Global Health Institute<br>Stanifer, John; Duke University, Division of Nephrology, Department of Medicine; Duke Global Health Institute
**Primary Subject Heading**:	Global health
Secondary Subject Heading:	Cardiovascular medicine, Qualitative research, Public health, Epidemiology
Keywords:	Hypertension < CARDIOLOGY, QUALITATIVE RESEARCH, health disparities, non-communicable diseases

SCHOLARONE™
Manuscripts

EPIDEMIOLOGY OF HYPERTENSION IN NORTHERN TANZANIA: A COMMUNITY-BASED MIXED-METHODS STUDY

CORRESPONDING AUTHOR:
Sophie W Galson, MD
310 Trent Drive, Duke Box 90518
Durham, NC 27708
Sophie.Galson@duke.edu
(301)-502-7718

AUTHORS:
Sophie W Galson, MD
Division of Emergency Medicine
Department of Surgery, Duke University; Durham, NC United States
Duke Global Health Institute

Catherine Staton, MD MSc
Division of Emergency Medicine
Department of Surgery, Duke University; Durham, NC United States
Division of Global Neurosurgery and Neuroscience, Department of Neurosurgery
Duke Global Health Institute

Francis Karia, MBA, MSc-PH
Kilimanjaro Christian Medical College; Moshi, Tanzania

Kajiru Kilonzo, MD, MMed
Department of Medicine, Kilimanjaro Christian Medical Center; Moshi, Tanzania

Joseph Lunyera, MMBS
Division of General Internal Medicine
Department of Medicine, Duke University; Durham, NC United States

Uptal D Patel, MD
Duke Clinical Research Institute
Department of Medicine, Duke University; Durham, NC United States

Julian T Hertz, MD
Division of Emergency Medicine
Department of Surgery, Duke University; Durham, NC United States
Duke Global Health Institute

John Stanifer, MD, MSc
Division of Nephrology
Department of Medicine, Duke University; Durham, NC United States
Duke Global Health Institute

WORD COUNT: 4,545

For peer review only

ABSTRACT

Introduction: Sub-Saharan Africa (SSA) is particularly vulnerable to the growing global burden of hypertension but epidemiological studies are limited and barriers to optimal management are poorly understood. Therefore, we undertook a community-based mixed-methods study in Tanzania to investigate the epidemiology of hypertension and barriers to care.

Methods: In northern Tanzania, between December 2013 and June 2015, we conducted a mixed-method study, including a cross-sectional household epidemiological survey and qualitative sessions of focus groups and in-depth interviews. For the survey, we assessed for hypertension, defined as a single blood pressure ≥ 160/100 mmHg or a two-time average of ≥ 140/90 mmHg or current use of anti-hypertensive medications. To investigate relationships with potential risk factors, we used adjusted generalized linear models. Uncontrolled hypertension was defined as a two-time average measurement of ≥ 160/100 mmHg irrespective of treatment status. Hypertension awareness was defined a self-reported disease history in a participant with confirmed hypertension. To explore barriers to care, we identified emerging themes using an inductive approach within the Framework method.

Results: We enrolled 481 adults (median age 45) from 346 households, including 123 men (25.6%) and 358 women (74.4%). Overall, the prevalence of hypertension was 28.0% (95% CI 19.4-38.7), which was independently associated with age >60 years (prevalence risk ratio [PRR] 4.68; 95% CI 2.25-9.74) and alcohol use (PRR 1.72 (95% CI 1.15-2.58)). Traditional medicine use was inversely associated with hypertension (PRR 0.37 (0.26, 0.54)). Nearly half (48.3%) of the participants were aware of their disease, but almost all (95.3%) had uncontrolled hypertension. In the qualitative sessions, we identified barriers to optimal care, including poor point-of-care communication; poor understanding of hypertension; and structural barriers such as long wait-times and under-trained providers.

Conclusions: In northern Tanzania, the burden of hypertensive disease is substantial and optimal hypertension control is rare. Trans-disciplinary strategies sensitive to local practices should be explored to facilitate early diagnosis and sustained care delivery.

Keywords: Hypertension, Qualitative research, Health Disparities, Non-communicable diseases

For peer review only

STRENGTHS AND LIMITATIONS OF THIS STUDY

- Rigorous study design based on random, community-based sampling.
- The mixed-methods approach allowed for triangulation from multiple data sources with reproducible methods.
- Barriers to optimal hypertension care were explored using qualitative studies with key informants from both biomedicine and traditional medicine practices.
- As this was a cross-sectional study, causal inferences cannot be drawn.
- Our role as biomedical practitioners may have limited our ability to interpret results (researcher bias) about differences in disease understanding, and our inferences are derived from a biomedical perspective.

INTRODUCTION

Non-communicable diseases (NCDs), including hypertension, are a global epidemic disproportionately affecting health outcomes in low-and middle-income countries (LMICs) [1-3]. In sub-Saharan Africa (SSA) alone, more than 125 million people are expected to have hypertension by the year 2025 [4, 5]. Hypertensive-related complications are currently one of the leading causes of morbidity in SSA, and by 2030 hypertension and other NCDs are projected to surpass communicable diseases as the top cause of mortality [2, 3, 5]. Despite the overwhelming burden, SSA is mostly unprepared to address this impending public health crisis [6, 7].

Previously-identified barriers to addressing hypertension in SSA include under-recognition, under-treatment, and a limited understanding of its epidemiology [8, 9]. In particular, the lack of reliable health statistics and a paucity of community-based epidemiological data limit the ability for detection, surveillance, and creation of public health strategies for prevention and treatment [7, 10]. In northern Tanzania, for example, only 10-20% of patients with previously detected hypertension are receiving treatment, and only 16% of those on treatment were adequately controlled [11, 12].

Differences in hypertension care are related to several factors beyond healthcare access alone, including limited health literacy, cultural and social barriers, and heuristically different health belief models [13]. As such, understanding the epidemiology of hypertension as well as the social and community barriers to optimal care is critical for developing prevention and treatment strategies; however, few such studies have been conducted. Therefore, as part of the Comprehensive Kidney Disease Assessment for Risk Factors, Epidemiology, Knowledge, and Attitudes (CKD-AFRiKA) study, we conducted a mixed-methods study in order to characterize the community-based epidemiology of hypertension and barriers to optimal care through exploration of patient- and community-centered perspectives [14, 15].

METHODS

Ethics Statement

The study protocol was approved by Duke University Institutional Review Board (#Pro00040784), the Kilimanjaro Christian Medical College (KCMC) Ethics Committee (EC#502), and the National Institute for Medical Research

(NIMR) in Tanzania. Written informed consent (by signature or thumbprint) was obtained from all participants, and all participants with abnormal findings received counseling, educational pamphlets, and reimbursement with referral for follow-up. All participants were reimbursed between 1,500 and 12,000 Tanzanian Shillings (approximately 0.75 - 5.00 United States Dollars) depending upon their distance of travel.

Study Setting

We conducted a mixed methods study between December 2013 and June 2015 in the Kilimanjaro Region of Tanzania. The adult regional population is greater than 900,000 people, and it has a female majority (58%)[16]. Almost 35% of the adult population lives in an urban setting, which is comparable to national estimates, and the HIV prevalence is 3-5% [16, 17]. The unemployment rate is 19%, and most people have only a primary education (77%) [18]. The median age, average household size, and occupation distribution are similar to national estimates [16-18]. The largest ethnic group is the Chagga tribe, and Swahili is the major language [16]. The region comprises seven districts; our study was conducted in the Moshi Urban and Moshi Rural districts, which were selected based on their representative populations and proximity to our research infrastructure [16].

Quantitative Sampling and Data Collection

Detailed sampling methods for the CKD-AFRiKA Study have been previously reported [14, 15, 19]. In brief, a three-stage cluster probability sampling method, stratified by urban and rural setting, was used to randomly select neighborhoods based on probability proportional to size. Within each selected neighborhood, a cluster site was determined using geographic points randomly generated by Arc Global Information Systems (ArcGIS), v10.2.2 (Environmental Systems Research Institute, Redlands, CA). From the cluster site, households were then randomly chosen based on both a coin-flip and die-rolling technique according to our established protocol [14].

All non-pregnant, community-dwelling adults (age ≥ 18 years old) from the selected households were recruited into the study. The sample size was designed to estimate the prevalence of chronic kidney disease with a precision of 5% when accounting for the cluster-design effect. To reduce non-response rates, a minimum of two additional visits were attempted during off-hours (evenings and weekends) as well as multiple phone calls using mobile phone numbers.

All data were collected using trained, local surveyors. Each participant completed a demographic and medical history survey, which included self-reported history of diabetes, hypertension, HIV, kidney disease, and heart disease (coronary, structural, or heart failure). If participants were receiving biomedical treatment in the form of medical therapy, specific drug information was collected. Women additionally gave a self-reported history for pregnancy or menstruation. Awareness was defined as giving a self-reported history of hypertension and subsequently testing positive for hypertension in our screening process.

Anthropometric data (including height, weight, and body mass index) were recorded for each participant. Normal weight was defined as a BMI of 20 to 24.9 kg/m^2. Overweight was defined as a BMI ≥25 kg/m^2 and obesity was defined as a BMI ≥30 kg/m^2. We measured blood pressure using the automated Omron HEM-712 sphygmomanometer (Omron Healthcare, Inc.; Bannockburn, IL) that has an adjustable cuff size. The machine was calibrated monthly during data collection. All participants were seated in an erect position with feet flat on the floor for a minimum of five minutes before measurements. Two measurements separated by > 5 minutes were then performed. Hypertension was defined as a single blood pressure measurement of greater than 160/100 mmHg, a two-time average measurement of greater than 140/90 mmHg, or current self-reported use of anti-hypertensive medications. Uncontrolled hypertension was defined as a two-time average measurement

of greater than 160/100 mmHg irrespective of treatment status. Tobacco and alcohol use were defined as self-reported current ongoing use, former use, or never used.

Qualitative Data Collection

To explore patient- and community-centered perspectives related to barriers in optimal hypertension care, we conducted focus group discussions (FGDs) and in-depth interviews in a central, easily accessible location. These sessions have been described previously [20]. In brief, we conducted FGDs and in-depth interviews with key informants from the community including well-adults from the general population, chronically-ill adults receiving care at the hospital medicine clinics, adults receiving care from traditional healers, adults purchasing traditional medicines from herbal vendors, traditional healers, herbal vendors, and medical doctors. Purposive sampling was used to recruit the key informants. We targeted men and women of all ages from urban and rural settings with different education levels and ethnicities. FGDs were held in a rented office space in Moshi Urban that was well-known and easily accessible to local residents and ensured privacy. Each FGD lasted between four and six hours including breaks. In-depth interviews were conducted at the same office space with the exception of the traditional healers and herbal vendors who were interviewed at their places of work; these sessions lasted one to two hours. All sessions were semi-structured, open-ended, and probing. The discussion guide was initially written in English and then translated to Swahili by an independent team. All sessions were moderated by a native, local member of our team (FK). All sessions were audio-recorded, and two note-takers transcribed and independently translated each session. A moderator then reviewed the transcripts to ensure accuracy. Debriefings were held after each session, and team meetings were again held following translation.

Quantitative Data Analysis

The mean and standard deviation (SD) or median and inter-quartile ranges (IQR) were reported for continuous variables. Prevalence estimates were sample-balanced using age- and gender-weights based on the 2012 urban and rural district-level census data. We used a Chi-squared test or Fisher's exact test to compare differences between groups. All p-values are two-sided at a 0.05 significance level. Quantitative data were analyzed using STATAv.14 (STATA Corp., College Station, TX).

A secondary aim of the analysis was to explore associations between hypertension and potential risk factors related to lifestyle. Crude and adjusted prevalence risk ratios (PRR) were estimated using generalized linear models with a log link, and we used Taylor Series linearization to account for the design effect on variance due to cluster sampling. Separate uni-variable and multi-variable models were fitted to hypertension status for each lifestyle-related variable including alcohol use, tobacco use, traditional medicine use, living in an urban environment, and overweight/obesity status. Models were adjusted for confounding factors potentially associated with hypertension and each potential risk factor, including age, gender, and ethnicity. We did not include education or occupation in our models due to *a priori* assumptions about their potential upstream causal association with lifestyle-related risk factors.

All quantitative data were collected on paper and then electronically entered into and managed using REDCap electronic data capture tools hosted at Duke University [21]. All data were verified after electronic data entry by an independent reviewer to ensure accuracy.

Qualitative Data Analysis

We conducted a thematic analysis of the qualitative data by applying an inductive approach to the framework method [22]. The approach was based, in part, on our previously-developed model which explored

For peer review only

determinants of traditional medicine use and biomedical healthcare utilization among individuals in Kilimanjaro with NCDs, including hypertension [20]. After data reduction, we performed open-coding of all transcripts. We used a 'cultural insider' (emic) and a 'cultural outsider' (etic) to independently code the data. The cultural insider was a native researcher living in the region (FK) and the cultural outsider was a researcher foreign to the region (JWS). Comparisons were made between each code set and areas of disagreement were discussed and resolved by revisiting the data. This approach allowed us to explore concepts that otherwise may have been overlooked or misinterpreted by either researcher individually. The qualitative coding, analytic memos, and corresponding matrices were stored and analyzed using NViVOv.10.0 (QRS International Pty Ltd, Melbourne, Australia). The codes were grouped together into categories, and we used a coding index to formulate connections and explore relationships.

For p

RESULTS

Table 1. Baseline Characteristics for the Quantitative Study

Variable (n, %)	Total (n=481)	Normotensive (n=332)	Hypertensive (n=149)	p-value
**Gender**				0.12
Male	123 (25.6%)	78 (23.5%)	45 (30.2%)	
Female	358 (74.4%)	254 (76.5%)	104 (69.8%)	
**Age**				<0.01
18-39 years old	172 (35.8%)	152 (45.8%)	20 (13.4%)	
40-59 years old	191 (39.7%)	132 (39.8%)	59 (39.6%)	
60+ years old	118 (24.5%)	48 (14.5%)	70 (47.0%)	
**Ethnicity**				0.40
Chagga	288 (59.9%)	193 (58.1%)	95 (63.7%)	
Pare	66 (13.7%)	51 (15.4%)	15 (10.1%)	
Sambaa	27 (5.6%)	20 (6.0%)	7 (4.7%)	
Other[§]	100 (20.8%)	68 (20.5%)	32 (21.5%)	
**Education**				<0.01
None	31 (6.4%)	11 (3.31%)	20 (13.4%)	
Primary	349 (72.6%)	246 (74.1%)	103 (69.1%)	
Secondary	74 (15.4%)	54 (16.3%)	20 (13.4%)	
Post-Secondary	27 (5.6%)	21 (6.3%)	6 (4.03%)	
**Occupation**				<0.01
Unemployed[#]	74 (15.4%)	55 (16.6%)	19 (12.8%)	
Farmer/Wage Earner	199 (41.4%)	135 (40.7%)	64 (43.0%)	
Small Business/Vendors	158 (32.8%)	121 (36.5%)	37 (24.8%)	
Professional[†]	50 (10.4%)	21 (6.3%)	29 (19.5%)	
**Lifestyle Practices**				
Ongoing tobacco use	50 (10.4%)	34 (10.2%)	16 (10.7%)	0.87
Ongoing alcohol use	198 (41.2%)	121 (36.4%)	77 (51.7%)	0.02
Traditional medicine use	272 (56.6%)	196 (59.0%)	76 (51.0%)	0.10
**Self-Reported Medical History**				
Diabetes	61 (12.7%)	29 (8.7%)	32 (21.5%)	<0.01
Hypertension	134 (28.0%)	62 (18.8%)	72 (48.3%)	<0.01
Stroke	8 (1.7%)	2 (0.6%)	6 (4.0%)	0.01
Heart Disease[*]	18 (3.7%)	7 (2.1%)	7 (4.7%)	0.08
Kidney Disease	14 (2.9%)	10 (3.0%)	4 (2.7%)	0.84

Table 2. Baseline Characteristics for the Qualitative Study

	FGD1	FGD2	FGD3	FGD4	FGD5	In-Depth Interviews
**Study Population**	Clinic Patients	General Population	Clinic Patients	General Population	Medical Doctors	Patients from Healers and Vendors
**Participants (N)**	15	12	16	12	4	11
**Gender**						
Male	0 (0%)	0(0%)	16(100%)	12(100%)	2(50%)	5(45%)
Female	15(100%)	12(100%)	0(0%)	0(0%)	2(50%)	6(55%)
Age range (years)	25-61	26-65	18-70	18-74	30-36	19-60
**Ethnicity**						
Chagga	11 (73%)	9 (75%)	11 (69%)	4 (33%)	2 (50%)	2 (18%)
Pare	2 (13%)	2 (17%)	2 (13%)	5 (42%)	0	0
Maasai	0	0	0	0	0	4 (36%)
Sambaa	1 (7%)	1 (8%)	1 (6%)	0	0	3 (27%)
Other*	1 (7%)	0	2 (13%)	3 (25%)	2 (50%)	2 (18%)
**Education**						
None	0	0	0	0	0	2 (18%)
Primary	11 (73%)	10 (83%)	10 (63%)	3 (25%)	0	4 (36%)
Secondary	3 (20%)	2 (17%)	5 (31%)	6 (50%)	0	1 (9%)
University	1 (7%)	0	1 (6%)	3 (25%)	4 (100%)	4 (36%)
**Occupation**						
Unemployed[#]	2 (13%)	4 (33%)	0	1 (8%)	0	3 (27%)
Student	0	0	4 (25%)	5 (42%)	0	0
Farmer/Wage Earner	4 (27%)	3 (25%)	8 (50%)	3(25%)	0	5 (45%)
Small Business	3 (20%)	2 (17%)	3 (19%)	2 (17%)	0	1 (9%)
Professional[†]	4 (27%)	3 (25%)	1 (6%)	1 (8%)	4(100%)	2 (18%)
**Religion**						
Roman Catholic	5 (33%)	5 (42%)	8 (50%)	1 (8%)	3 (75%)	7 (64%)
Lutheran	6 (40%)	4 (33%)	4 (25%)	2 (17%)	0	1 (9%)
Christian Evangelical	1 (7%)	1 (8%)	2 (13%)	5 (42%)	1 (25%)	1 (9%)
Christian (Other)	2 (13%)	0	0	0	0	0
Islam	1 (7%)	2 (17%)	2 (13%)	4 (33%)	0	2 (18%)
**Residence**						
Urban	9 (60%)	11 (92%)	10 (83%)	12 (100%)	4 (100%)	9 (82%)
Rural	6 (40%)	1 (8%)	2 (17%)	0 (0%)	0 (0%)	2 (18%)

Study Populations

We enrolled 481 adults into the quantitative study (**Table 1**). The median age was 45.0 years (IQR 35–59). The majority of participants were women (n = 358; 74%), lived in an urban location (n = 370; 77%), ethnically Chagga (n = 288; 60%), and only had a primary school education (n = 349; 73%). The most common occupation among participants was farming or daily wage work (n =199; 41%). Many participants reported ongoing use of alcohol (n=198; 41%) and traditional medicine use over the previous year (n=272; 57%), with the most commonly reported frequency of traditional medicine use at 1–5 times (31.0 %) per year. Among participants currently using prescribed biomedicines (n=70), the proportion of participants reporting traditional medicine use was more substantial, with 69% (n=48) reporting traditional medicine use over the previous year.

The household non-response rate was 15.0% and the individual non-response rate was 20.6%. Compared with the regional population [16], men (p<0.001) and young adults 18–39 years old (p = 0.001) were more likely to be non-responders in our study, and the proportion of participants with a secondary or post-secondary education

(22%) was higher than the regional average (15%) (p = 0.02). We observed no significant differences in occupation between the responders and non-responders (p = 0.64).

In the qualitative study, we conducted five FGDs and 11 in-depth interviews (**Table 2**). FGDs and in-depth interviews included even numbers of men (n= 35; 50%), and women (n=35; 50%) and had an age range of 18 to 74 years. Most participants were of the Chagga ethnic group (n=37; 53%), and were Roman Catholic (n=29; 41%), but Islamic (n=11; 16%), Lutheran (n=17; 24%), and Christian Evangelical (n=11; 16%) were also represented as well as thirteen different tribal ethnicities. Education levels varied from none (n=2; 3%) to university level (n=13; 19%), but the majority had only completed a primary education (n=38; 54%). Most participants were from urban residences (n=55; 79%).

Burden of Hypertension

The prevalence of hypertension was 28% (95% CI 19.4-38.7). The design effect of the cluster sampling was 2.34, with a neighborhood-level ICC coefficient of 0.075. Mean systolic blood pressure was 129.5 mmHg (SD 24.3), mean diastolic blood pressure was 77.6 mmHg (SD 12.2), and the median age was 58 years (IQR 45-65). The prevalence of hypertension was 59.3% (95% CI 50.1-67.9) in participants ≥ 60 years old. Comparatively, prevalence was 30.9% (95% CI 24.7-37.9) in the 40-59 age group and 11.6% (95% CI 7.6-17.4) in the under 40-age group. The median BMI of individuals with hypertension was 27.4 kg/m^2 (IQR 24-30). Participants with hypertension were more likely to be men, older, report ongoing alcohol use, live in an urban environment, have less education, and be employed as professionals (p<0.05 for all) (**Table 1**). The proportion of reported traditional medicine use among participants with hypertension was 39.3% (95% CI 30.3-49.1%), and the most common reasons reported for using traditional medicines were to treat daily symptomatic ailments (45%) and for treatment of chronic diseases (10%), including hypertension (**Figure 1**).

Crude and adjusted prevalence risk ratios for the relation between lifestyle-related factors and hypertension are reported in **Table 3**. In crude models, traditional medicine use was inversely associated with hypertension prevalence (PRR 0.60; 95% CI 0.41-0.87), and alcohol use was significantly associated with higher prevalence of hypertension (PRR 2.29; 95% 1.26-4.15). These associations remained significant even after adjustment for age, gender, and ethnicity, with a PRR 0.37 (95% CI 0.26-0.54) and PRR 1.72 (95% CI 1.15-2.58) for traditional medicine use and alcohol use, respectively. We did not observe an association between hypertension prevalence and obesity, urban residence, or tobacco use (p>0.05 for all).

Table 3. Associations between lifestyle factors and hypertension; CKD-AFRIKA, 2015.

Variables	Prevalence Risk Ratios (95% CI)	
	**Unadjusted**	**Adjusted***
**Ongoing Tobacco Use**	1.25 (0.75, 2.10)	0.68 (0.41, 1.14)
**Traditional Medicine Use**	**0.60 (0.41, 0.87)**	**0.37 (0.26, 0.54)**
**Ongoing Alcohol Use**	**2.29 (1.26, 4.15)**	**1.72 (1.15, 2.58)**
**Overweight/Obese**	1.00 (0.57, 1.74)	1.28 (0.84, 1.97)
**Urban Residence**	0.58 (0.34, 1.00)	0.85 (0.59, 1.22)

***Adjusted for age, gender, and ethnicity**
BOLD: Significant at the 5% level

Barriers to Optimal Care

Despite the high disease burden, only half of participants with elevated blood pressure (48%) were aware of having hypertension. Few (23%) reported taking biomedicines for hypertension, and 12% reported taking both biomedicines and traditional medicines. Almost all participants (95%) had uncontrolled hypertension. A major theme that emerged as an important barrier for awareness and disease self-management was a difference in individual chronic disease understanding. Most notably, we identified quality or perceived quality of the biomedical healthcare delivery, disease expression, chronicity of disease, and traditional health belief models as important contributors to the observed differences in chronic disease understanding, which itself contributed to unrealistic expectations of cure, perceived treatment failures, and medical non-compliance (**Figure 2**).

Participants related structural issues and point-of-care communication issues as key barriers to optimal biomedical healthcare delivery. Structural issues in the delivery of biomedical healthcare, including long wait times, under-staffing, lack of experience by healthcare providers, and medication costs, were particularly concerning, and together with poor point-of-care communication between patient and provider, appeared to substantially contribute to differences in chronic disease understanding through unrealistic expectations of cure expressed as concern over ineffective or inappropriate therapies:

> *MDs have a lot of patients to take care of so they lack enough time to give explanations. They hurry so that they can save as many patients as possible.*
>
> *I attended the hospital and treatment was begun but with no success for a long time; instead other parts in my body began to swell. At that time I decided to discharge myself from the hospital, and my grandmother gave me local herbals which cured me.*

For many participants, differences in disease understanding were also closely related to the disease expression or the symptom complex of disease. For chronic, generally asymptomatic diseases such as hypertension, this led to unrealistic expectations of cure, greater perceptions of treatment failure by biomedicines, and increased medical non-compliance:

> *You know you have a disease because the body always has symptoms.*
>
> *You know you are healed as you do not have to attend the hospital anymore because your symptoms have disappeared.*

For chronic diseases such as hypertension, chronicity or duration of disease was an especially salient topic closely related to differences in disease understanding. Chronic diseases were understood to be diseases that have either been untreated or undertreated, and even infectious diseases were viewed as chronic when left untreated:

> *Anything that stays in the human body for a long time without being cured, like amoebae and bilharzias* [schistosomiasis] *is a chronic disease.*

For peer review only

From the biomedical perspective, this different understanding of chronicity led to many challenges in achieving optimal care for patients with hypertension, particularly with respect to medication compliance, expectations of cure, and perceived treatment failure. As biomedical doctors explained:

> *Some patients with hypertension, after their blood pressure is controlled, they then believe they are cured after 2 or 3 months. They go to follow-up and see that their blood pressure normal; therefore, they assume they are cured and stop their medications. The chronicity of the disease they do not understand well.*

> *…the people keep on seeking a 'cure' for something that is a chronic disease.*

Finally, traditional health belief models were also closely associated with differences in disease understanding. Even for chronic diseases such as hypertension, participants expressed the importance of traditional medicines, and community elders and family members were considered important sources of healthcare knowledge.

> *My family and I prefer not to go to hospitals. My grandparents taught us a lot (especially about plant roots) about healing and curing… my father still will not use any hospital medicines.*

> *…most of the chronic diseases are cured by traditional medicines.*

Discussion

In a community-based setting in northern Tanzania, we found an alarmingly high burden of hypertension. The prevalence observed was 10-20% higher than similar regions in SSA and comparable with several upper and middle-income countries, including South Africa, Brazil, and China [2, 7, 9, 12, 23-25]. Despite the high burden of hypertension, awareness was low and few had achieved optimal blood pressure control, which may be explained by observed gaps in communication, quality of healthcare delivery, and traditional medicine health belief models. In particular, we identified differences in disease understanding as they relate to a disease expression through symptom complexes, disease chronicity, and traditional health beliefs as potentially important barriers for achieving optimal hypertension care.

As epidemiological transitions reshape the region, Tanzania is at great risk for an explosive growth in the burden of NCDs, including hypertension [7, 26, 27]. The rapid pace of urbanization and economic growth are accelerating the rate of this transition; thus, as evidenced by the high prevalence we are already observing, there is an urgent need for action [24]. Aggressive efforts should be made to diagnose and capture hypertensive patients at every single interaction within health systems. Considering the low rates of awareness in our study, all settings for diagnosis and delivery of healthcare should be explored including community centers and traditional medicine providers. Community-centered care models may be beneficial to reduce risk factors, improve treatment adherence, and have been successful previously in SSA [6, 28]. Given the important role of traditional medicine practices and prior willingness to refer patients to biomedical facilities for diagnostic testing [29], partnership with traditional medicine providers should be considered to assist with risk factor reduction and care coordination, including early referral [29, 30].

Additionally, emergency department (ED)-based screening has also been successful at capturing undiagnosed/uncontrolled hypertension cases and linking patients to care in high-income countries; yet ED-

For peer review only

based care models for hypertension have not been widely explored in SSA [31-33]. For example, ED-based interventions to screen for hypertension and modifiable risk factors as the first step of a care pathway may prove to be highly effective in hypertension care. Beyond diagnosis, careful consideration of the local environment and barriers to care will be necessary to create successful educational programs and sustain hypertension control. Educational interventions should focus on the concepts of chronicity and disease expression, and incorporate traditional health beliefs. Targeted and culturally-tailored engagement with patients may prevent poor hypertension outcomes through improved self-management, particularly with medication compliance, expectations of cure, and perceived treatment success [34].

The application of locally-tailored programs will also require a comprehensive understanding of traditional medicine practices, particularly as they intersect with biomedical concepts of health. We found traditional medicines use was substantially higher but associated with a lower prevalence of hypertension, and more work is needed to understand whether this could indicate a protective effect or alternatively, a form of selection bias, reporting bias, or unmeasured confounding. We also identified high concurrent use of traditional medicines and biomedicines, which further stresses the importance of culturally competent interventions when addressing hypertension. Previous research by our group in the region found that traditional medicines are used by 50%-70% of the general population, consistent with other reports across Tanzania [20, 35, 36]. Specifically, in the current study, among those with hypertension reporting the ongoing use of biomedicine, over 60% of participants were concurrently using traditional medicines, spanning all incomes, education levels and residential settings. Although we previously identified 168 separate plant-based traditional medicines used in this region, including two used specifically for treatment of hypertension (Lemongrass (*Cymbopogon citrullus)* and Erabel *(scientific name unknown),* further research is greatly needed to classify traditional plant-based-medicines, including their mechanisms of action, side effect profiles, and potential protective effects.

In contrast to traditional medicines, we found alcohol to be positively associated with hypertension in this population, consistent with trends throughout SSA [37-39]. This region of Tanzania has a particularly high prevalence of alcohol use due to widespread cultural acceptability and home-brewing culture [40]. Given the high burden of alcohol, practitioners in all locations should be cognizant of this risk factor during both hypertension screening and treatment. Conversely, hypertension prevalence was not associated with obesity, urban residence or tobacco use, all well-established risk factors in high-income countries [38, 41]. While prevalence was high in both rural and urban settings, it is unclear what factors may contribute to the chronic disease burdens across SSA and the drivers of hypertension may be different in urban vs. rural settings (e.g. dietary changes, environmental exposures, or differential access to care) [42, 43]. As with other chronic diseases, the link between obesity and hypertension is also not well understood in sub-Saharan Africa [44], and it is currently unclear to what extent genetic, lifestyle, and environmental factors interact to drive hypertension disparities [26, 45]. As such, future work investigating the determinants and risk factors for hypertension in this setting is urgently needed.

Our study was unique in exploring hypertension in the Kilimanjaro area by utilizing a rigorous randomly-sampled, household-level survey as part of a mixed-method design that also included qualitative sessions with key informants. We explored latent themes and social/community context for treatment failure, and by leveraging this thematic analysis we were able to identify targetable barriers to optimal hypertension diagnosis, treatment, and control. Nonetheless, we noted potential limitations to our study. First, selection bias from non-response may be present. To address any non-response bias that may have arisen from differences between the respondents and non-respondents, we used sample-balanced weights for age and gender and explored differences in occupation and education level between the two groups. In regards to internal validity, we only measured blood pressure at one setting; however, two measurements separated by > 5 minutes were

For peer review only

performed and previous studies have shown that sustained elevated blood pressure in one setting may be sensitive to establish a diagnosis of hypertension [32]. Misclassification of disease around the cutoff points for hypertension may also be present although we expect this misclassification to be non-differential. Medical history was also determined by self-report for several conditions, which may be less accurate in this setting with low awareness. Also, alcohol and tobacco use were not quantified and future research would be strengthened by quantifying tobacco and alcohol use with validated instruments [46]. Additionally, as this was a cross-sectional study, causal inferences cannot be drawn and associations may be influenced by confounding from unmeasured variables. Our role as biomedical practitioners may have limited our ability to interpret results (researcher bias) about differences in disease understanding, and our inferences are derived from a biomedical perspective. Finally, although we used insider-outsider coding, local non-medical surveyors, and local moderators for qualitative data collection, reporting bias may still be present.

In conclusion, in a community-based study of adults from the Kilimanjaro region of northern Tanzania we observed a high prevalence of hypertension, most of which was uncontrolled. Alcohol use may be an important risk factor for hypertension, and we identified several emerging cultural and social themes as barriers to optimal hypertension care, including most notably difference in disease understanding related to quality of healthcare delivery, chronicity of disease, disease expression, and traditional belief models. Hypertension care-models will need to leverage all existing resources from the ED, to community centers, to traditional healers in order to address the growing burden of hypertension in the region, and future studies are needed to develop targeted, culturally-tailored interventions designed to improve hypertension disease understanding.

ACKNOWLEDGMENTS

We would like to thank Professors G. Ralph Corey and John Bartlett, and all the staff of the KCMC-Duke Collaboration in Moshi, Tanzania for all of their efforts on the Comprehensive Kidney Disease Assessment For Risk factors, epidemiology, Knowledge, and Attitudes (CKD AFRiKA) Study. We give a special thanks to Carol Sangawe, Cynthia Asiyo, Nicola West, and Edith Aloyce Macha for their integral role implementing the study and Jeffrey Hawley and Audrey Brown at the Duke Office of Clinical Research for their help in data management. We are also grateful to Estomih Mduma and his team for their help with Swahili translation needs.

CONTRIBUTION

JS and UP developed the concept of the project. JS and FK conducted data collection. SG, JS, and JL contributed to writing the manuscript. JS and JL were responsible for the analysis plan and data analysis. CS, JS, SG, JL, KK, JH, UP, and FK were responsible for the final editing and all authors approved the final manuscript.

COMPETING INTERESTS

All authors have completed the ICMJE uniform disclosure form at www.icmje.org/coi_disclosure.pdf and declare: no support from any organization for the submitted work; no financial relationships with any organizations that might have an interest in the submitted work in the previous three years; no other relationships or activities that could appear to have influenced the submitted work.

FUNDING

This study was supported by an NIH Research Training Grant (#R25 TW009337) funded by the Fogarty International Center and the National Institute of Mental Health; and a Research and Prevention Grant funded

For peer review only

by the International Society of Nephrology Global Outreach Committee. Dr. Staton would like to acknowledge salary support funding from the Fogarty International Center (Staton, K01 TW010000-01A1).

DATA SHARING

We have concerns about the ethics of openly releasing the entire dataset to the public as the structure of the dataset would result in loss of participant anonymity. However, we will ensure that the dataset is openly available to researchers who contact us and meet confidentiality requirements (documentation of ethics training in conduct of human-subject research). They may contact Dr. John W Stanifer, DCRI 2400 Pratt St, Durham NC 27710 or john.stanifer@duke.edu

FIGURE LEGEND

Fig. 1 Reported reasons for using traditional medicines among participants with hypertension.

Fig. 2 Conceptual model describing the hypothesized relationship between disease understanding and hypertension outcomes.

REFERENCES

1. Abegunde DO, Mathers CD, Adam T, et al. The burden and costs of chronic diseases in low-income and middle-income countries. *Lancet (London, England)*. 2007;370(9603):1929-38.
2. WHO | A global brief on hypertension. *WHO*. 2013.
3. Institute for Health Metrics and Evaluation (IHME) 2015 [Available from: http://www.healthdata.org/].
4. Pastakia SD, Ali SM, Kamano JH, et al. Screening for diabetes and hypertension in a rural low income setting in western Kenya utilizing home-based and community-based strategies. *Globalization and health*. 2013;9:21.
5. Whitworth JA. 2003 World Health Organization (WHO)/International Society of Hypertension (ISH) statement on management of hypertension. *Journal of hypertension*. 2003;21(11):1983-92.
6. Twagirumukiza M, Van Bortel LM. Management of hypertension at the community level in sub-Saharan Africa (SSA): towards a rational use of available resources. *Journal of human hypertension*. 2011;25(1):47-56.
7. Peck R, Mghamba J, Vanobberghen F, et al. Preparedness of Tanzanian health facilities for outpatient primary care of hypertension and diabetes: a cross-sectional survey. *Lancet Glob Health*. 2014;2(5):e285-92.
8. Naanyu V, Vedanthan R, Kamano JH, et al. Barriers Influencing Linkage to Hypertension Care in Kenya: Qualitative Analysis from the LARK Hypertension Study. *Journal of general internal medicine*. 2016;31(3):304-14.
9. Chow CK, Teo KK, Rangarajan S, et al. Prevalence, awareness, treatment, and control of hypertension in rural and urban communities in high-, middle-, and low-income countries. *Jama*. 2013;310(9):959-68.
10. Churchill LO. Epidemiology of ischaemic heart disease in sub-Saharan Africa. *Cardiovasc J Afr*. 2013;24(2):34-42.
11. Dewhurst MJ, Dewhurst F, Gray WK, et al. The high prevalence of hypertension in rural-dwelling Tanzanian older adults and the disparity between detection, treatment and control: a rule of sixths? *Journal of human hypertension*. 2012;27(6):374-80.
12. Mosha NR, Mahande M, Juma A, et al. Prevalence,awareness and factors associated with hypertension in North West Tanzania. *Global health action*. 2017;10(1):1321279.

For peer review only

13. Nnko S, Bukenya D, Kavishe BB, et al. Chronic Diseases in North-West Tanzania and Southern Uganda. Public Perceptions of Terminologies, Aetiologies, Symptoms and Preferred Management. *PloS one*. 2015;10(11):e0142194.
14. Stanifer JW, Maro V, Egger J, et al. The epidemiology of chronic kidney disease in Northern Tanzania: a population-based survey. *PloS one*. 2015;10(4):e0124506.
15. Stanifer JW, Egger JR, Turner EL, et al. Neighborhood clustering of non-communicable diseases: results from a community-based study in Northern Tanzania. *BMC Public Health*. 2016;16:226.
16. Central Census Office and National Bureau of Statistics. United Republic of Tanzania: 2012 Population and Housing Census 2013 [Available from: http://www.nbs.go.tz. .
17. Demographic and Health Surveys. Program. United Republic of Tanzania HIV/AIDS and Malaria Indicator Survey 2011-2012. 2013 [Available from: http://dhsprogram.com/pubs/pdf/AIS11/AIS11.pdf.
18. Development Committee. United Republic of Tanzania: Education Sector Performance Report, 2010-2011 2013 [Available from: http://www.ed-dpg.or.tz.
19. Stanifer JW, Cleland CR, Makuka GJ, et al. Prevalence, Risk Factors, and Complications of Diabetes in the Kilimanjaro Region: A Population-Based Study from Tanzania. *PloS one*. 2016;11(10):e0164428.
20. Stanifer JW, Patel UD, Karia F, et al. The determinants of traditional medicine use in Northern Tanzania: a mixed-methods study. *PloS one*. 2015;10(4):e0122638.
21. Harris PA, Taylor R, Thielke R, et al. Research electronic data capture (REDCap)--a metadata-driven methodology and workflow process for providing translational research informatics support. *Journal of biomedical informatics*. 2008;42(2):377-81.
22. Gale NK, Heath G, Cameron E, et al. Using the framework method for the analysis of qualitative data in multi-disciplinary health research. *BMC medical research methodology*. 2013;13:117.
23. Pastakia SD, Ali SM, Kamano JH, et al. Screening for diabetes and hypertension in a rural low income setting in western Kenya utilizing home-based and community-based strategies. Globalization and health. 92013. p. 21.
24. Twagirumukiza M, De Bacquer D, Kips JG, et al. Current and projected prevalence of arterial hypertension in sub-Saharan Africa by sex, age and habitat: an estimate from population studies. *Journal of hypertension*. 2011;29(7):1243-52.
25. Kearney PM, Whelton M, Reynolds K, et al. Worldwide prevalence of hypertension: a systematic review. *Journal of hypertension*. 2004;22(1):11-9.
26. Yikona J. Non-communicable disease in sub-Saharan Africa. *Lancet (London, England)*. 2001;357(9249):74.
27. Mufunda J, Chatora R, Ndambakuwa Y, et al. Emerging non-communicable disease epidemic in Africa: preventive measures from the WHO Regional Office for Africa. *Ethnicity & disease*. 2006;16(2):521-6.
28. Jeffries D. Risk in cardiovascular disease. Having so many different guidelines about reducing risk is confusing. *BMJ (Clinical research ed)*. 2000;321(7254):175.
29. Liwa AC, Smart LR, Frumkin A, et al. Traditional Herbal Medicine Use Among Hypertensive Patients in Sub-Saharan Africa: A Systematic Review. *Curr Hypertens Rep*. 2014;16(6):437.
30. Gessler MC, Msuya DE, Nkunya MH, et al. Traditional healers in Tanzania: sociocultural profile and three short portraits. *Journal of ethnopharmacology*. 1995;48(3):145-60.
31. Chernow SM, Iserson KV, Criss E. Use of the emergency department for hypertension screening: a prospective study. *Annals of emergency medicine*. 1987;16(2):180-2.
32. Ackerson HDB, Linda D, Lynn. Reproducibility of increased blood pressure during an emergency department or urgent care visit. *Annals of emergency medicine*. 2003;41(4):507-12.
33. Tan N, Taylor DM. Feasibility and outcomes of screening for cardiovascular risk factors in the emergency department. *Emergency medicine Australasia : EMA*. 2013;25(2):175-81.

For peer review only

34. Warsi A WP, LaValley MP, Avorn J, Solomon DH. Self-management education programs in chronic disease: a systematic review and methodological critique of the literature. *Arch Intern Med*. 2004:9-23;164(15):1641-9.
35. Kayombo EJ, Uiso FC, Mahunnah RL. Experience on healthcare utilization in seven administrative regions of Tanzania. *Journal of Ethnobiology and Ethnomedicine*. 2012;8(1):5.
36. Liwa AC, Smart LR, Frumkin A, et al. Traditional Herbal Medicine Use Among Hypertensive Patients in Sub-Saharan Africa: A Systematic Review. *Current Hypertension Reports*. 2014;16(6):437.
37. Xin X, He J, Frontini MG, et al. Effects of alcohol reduction on blood pressure: a meta-analysis of randomized controlled trials. *Hypertension*. 2001;38(5):1112-7.
38. He J, Bazzano LA. Effects of lifestyle modification on treatment and prevention of hypertension. *Current opinion in nephrology and hypertension*. 2000;9(3):267-71.
39. Nahimana MR, Nyandwi A, Muhimpundu MA, et al. A population-based national estimate of the prevalence and risk factors associated with hypertension in Rwanda: implications for prevention and control. *BMC Public Health*. 2017;18(1):2.
40. Mitsunaga T, Larsen U. Prevalence of and risk factors associated with alcohol abuse in Moshi, northern Tanzania. *Journal of biosocial science*. 2008;40(3):379-99.
41. Pereira M, Lunet N, Azevedo A, et al. Differences in prevalence, awareness, treatment and control of hypertension between developing and developed countries. *Journal of hypertension*. 2009;27(5):963-75.
42. Njelekela M, Sato T, Nara Y, et al. Nutritional variation and cardiovascular risk factors in Tanzania--rural-urban difference. *South African medical journal = Suid-Afrikaanse tydskrif vir geneeskunde*. 2003;93(4):295-9.
43. Damasceno A, Azevedo A, Silva-Matos C, et al. Hypertension prevalence, awareness, treatment, and control in mozambique: urban/rural gap during epidemiological transition. *Hypertension*. 2009;54(1):77-83.
44. Okosun IS, Rotimi CN, Forrester TE, et al. Predictive value of abdominal obesity cut-off points for hypertension in blacks from west African and Caribbean island nations. *International journal of obesity and related metabolic disorders : journal of the International Association for the Study of Obesity*. 2000;24(2):180-6.
45. Mathenge W, Foster A, Kuper H. Urbanization, ethnicity and cardiovascular risk in a population in transition in Nakuru, Kenya: a population-based survey. *BMC Public Health*. 2010;10:569.
46. Lundin A, Hallgren M, Balliu N, et al. The use of alcohol use disorders identification test (AUDIT) in detecting alcohol use disorder and risk drinking in the general population: validation of AUDIT using schedules for clinical assessment in neuropsychiatry. *Alcoholism, clinical and experimental research*. 2015;39(1):158-65.

For peer review only

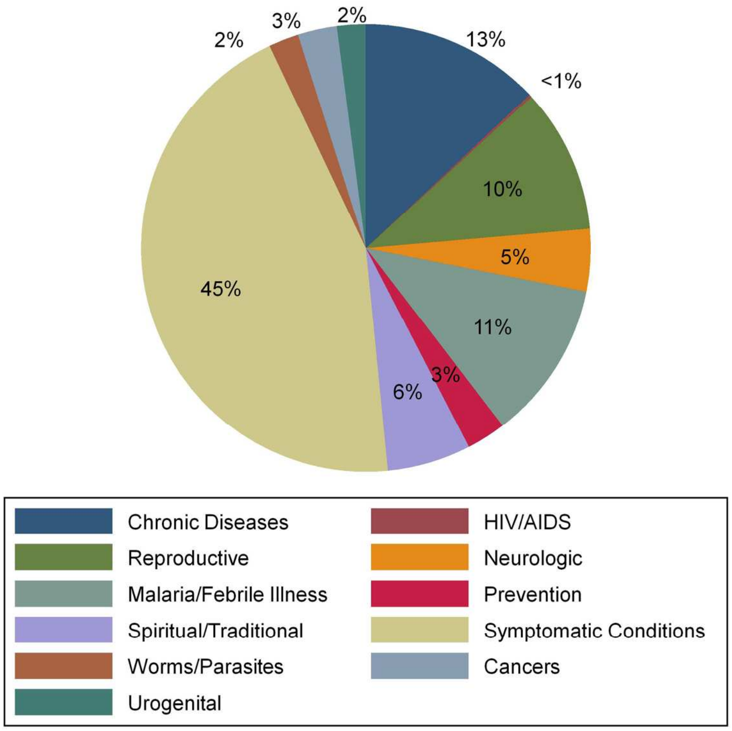

Figure 1. Reported reasons for using traditional medicines among participants with hypertension

86x64mm (600 x 600 DPI)

For peer review only - http://bmjopen.bmj.com/site/about/guidelines.xhtml

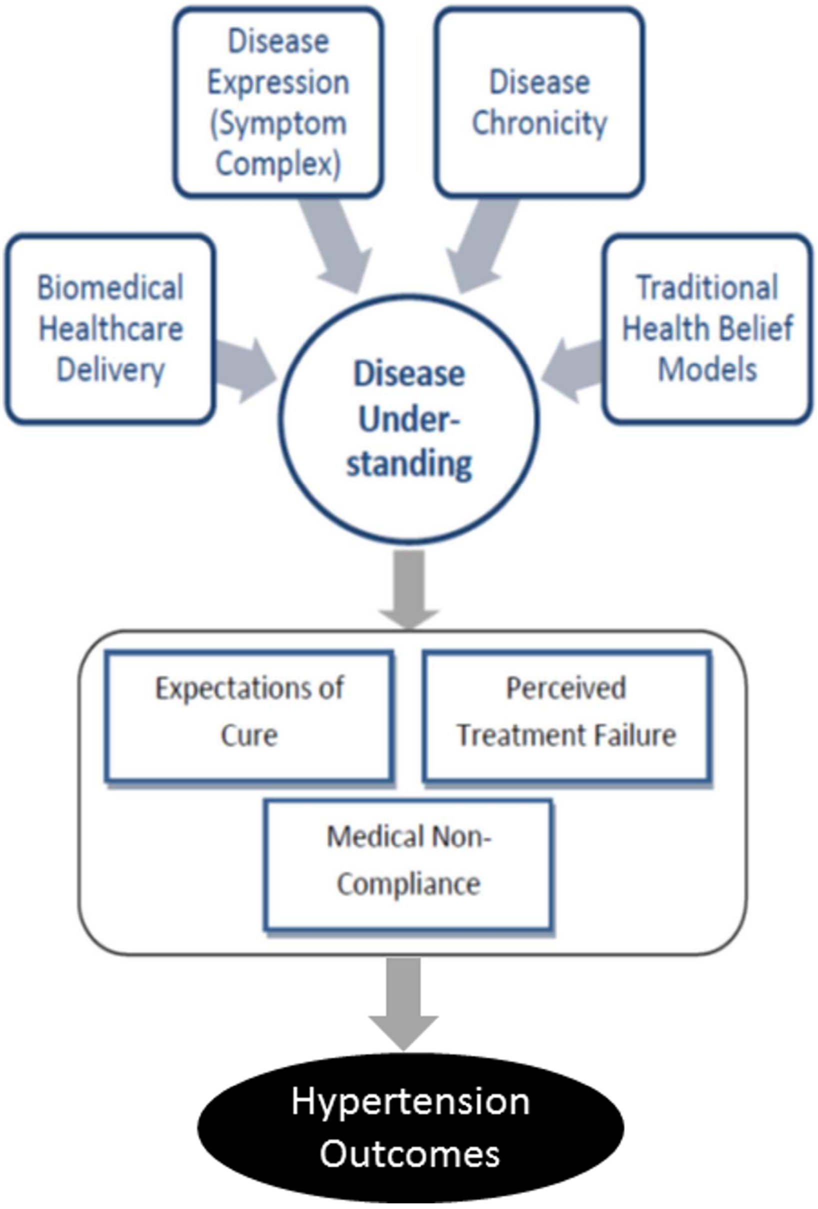

Figure 2. Conceptual model describing the hypothesized relationship between disease understanding and hypertension outcomes

189x279mm (300 x 300 DPI)

STROBE 2007 (v4) Statement—Checklist of items that should be included in reports of *cross-sectional studies*

Section/Topic	Item #	Recommendation	Reported on page #
**Title and abstract**	1	(*a*) Indicate the study's design with a commonly used term in the title or the abstract	1
		(*b*) Provide in the abstract an informative and balanced summary of what was done and what was found	2
**Introduction**			
Background/rationale	2	Explain the scientific background and rationale for the investigation being reported	3
Objectives	3	State specific objectives, including any prespecified hypotheses	3
**Methods**			
Study design	4	Present key elements of study design early in the paper	4
Setting	5	Describe the setting, locations, and relevant dates, including periods of recruitment, exposure, follow-up, and data collection	4
Participants	6	(*a*) Give the eligibility criteria, and the sources and methods of selection of participants	4
Variables	7	Clearly define all outcomes, exposures, predictors, potential confounders, and effect modifiers. Give diagnostic criteria, if applicable	4
Data sources/ measurement	8*	For each variable of interest, give sources of data and details of methods of assessment (measurement). Describe comparability of assessment methods if there is more than one group	4
Bias	9	Describe any efforts to address potential sources of bias	*4*
Study size	10	Explain how the study size was arrived at	4
Quantitative variables	11	Explain how quantitative variables were handled in the analyses. If applicable, describe which groupings were chosen and why	5
Statistical methods	12	(*a*) Describe all statistical methods, including those used to control for confounding	5
		(*b*) Describe any methods used to examine subgroups and interactions	5
		(*c*) Explain how missing data were addressed	5
		(*d*) If applicable, describe analytical methods taking account of sampling strategy	5
		(*e*) Describe any sensitivity analyses	NA
**Results**			

For peer review only

Participants	13*	(a) Report numbers of individuals at each stage of study—eg numbers potentially eligible, examined for eligibility, confirmed eligible, included in the study, completing follow-up, and analysed	4
		(b) Give reasons for non-participation at each stage	4
		(c) Consider use of a flow diagram	4 (reference)
Descriptive data	14*	(a) Give characteristics of study participants (eg demographic, clinical, social) and information on exposures and potential confounders	6
		(b) Indicate number of participants with missing data for each variable of interest	4
Outcome data	15*	Report numbers of outcome events or summary measures	6,7
Main results	16	(*a*) Give unadjusted estimates and, if applicable, confounder-adjusted estimates and their precision (eg, 95% confidence interval). Make clear which confounders were adjusted for and why they were included	6,7
		(*b*) Report category boundaries when continuous variables were categorized	4
		(*c*) If relevant, consider translating estimates of relative risk into absolute risk for a meaningful time period	NA
Other analyses	17	Report other analyses done—eg analyses of subgroups and interactions, and sensitivity analyses	8
**Discussion**			
Key results	18	Summarise key results with reference to study objectives	10
Limitations	19	Discuss limitations of the study, taking into account sources of potential bias or imprecision. Discuss both direction and magnitude of any potential bias	11
Interpretation	20	Give a cautious overall interpretation of results considering objectives, limitations, multiplicity of analyses, results from similar studies, and other relevant evidence	10
Generalisability	21	Discuss the generalisability (external validity) of the study results	11
**Other information**			
Funding	22	Give the source of funding and the role of the funders for the present study and, if applicable, for the original study on which the present article is based	11

*Give information separately for cases and controls in case-control studies and, if applicable, for exposed and unexposed groups in cohort and cross-sectional studies.

Note: An Explanation and Elaboration article discusses each checklist item and gives methodological background and published examples of transparent reporting. The STROBE checklist is best used in conjunction with this article (freely available on the Web sites of PLoS Medicine at http://www.plosmedicine.org/, Annals of Internal Medicine at http://www.annals.org/, and Epidemiology at http://www.epidem.com/). Information on the STROBE Initiative is available at www.strobe-statement.org.

For peer review only
